# Design, Synthesis, and Temperature-Driven Molecular Conformation-Dependent Delayed Fluorescence Characteristics of Dianthrylboron-Based Donor–Acceptor Systems

**DOI:** 10.3389/fchem.2020.541331

**Published:** 2020-10-09

**Authors:** Umesh Pratap Pandey, Rajendra Prasad Nandi, Pakkirisamy Thilagar

**Affiliations:** Department of Inorganic and Physical Chemistry, Indian Institute of Science, Bangalore, India

**Keywords:** boron, aminoboron, TADF, anthracene, phenothiazine

## Abstract

We report a simple and novel molecular design strategy to enhance rISC in boron-based donor–acceptor systems to achieve improved delayed fluorescence characteristics. Dianthrylboryl ((An)_2_B)-based aryl aminoboranes **1** (donor: phenothiazine) and **2** (donor: N,N-diphenylamine) were synthesized by a simple one-pot procedure. The energy of the electronic excited states in **1** and **2** were modulated by varying the arylamine donor strength and electronic coupling between D and A moieties. The presence of a large π-system (anthryl moiety) on boron enhances the electronic communication between donor arylamine and acceptor boryl moieties, and hence, both **1** and **2** exhibit delayed fluorescence characteristics in a broad range of temperatures (80–300 K). Single crystal X-ray analysis and temperature-dependent photophysical studies together with theoretical studies were carried out to rationalize the observed intriguing optical signatures of **1** and **2**.

**Graphical Abstract:**
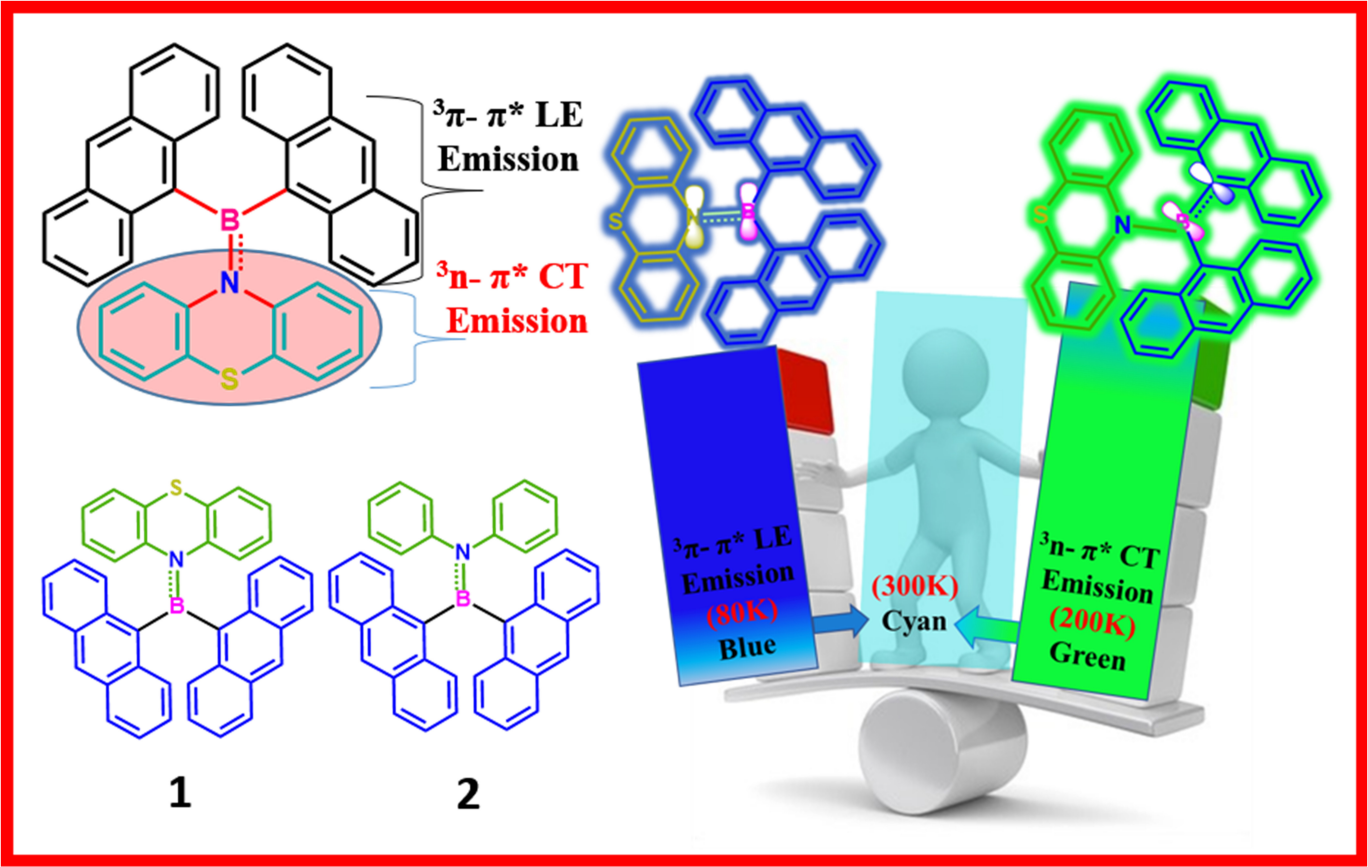


## Introduction

Improving the efficiency of optoelectronic devices by harvesting both triplet and singlet excitons is a contemporary area of research (Uoyama et al., 2012; Obolda et al., 2016; Noda et al., 2018; Liang et al., 2019; Northey et al., 2019). Various approaches such as triplet–triplet annihilation (TTA) (Yang et al., 2017; Pu et al., 2019; Ye et al., 2019), exciton–polaron interaction (EPI) (Kim et al., 2018), and thermally activated delayed fluorescence (TADF) (Uoyama et al., 2012) have been put forward for this application. This is because both TTA and EPI are bimolecular processes and it is very challenging to control these processes in devices. On the other hand, TADF is a temperature-driven unimolecular process and the device physics is less complicated compared to that of TTA and EPI. The temperature-dependent rate of reverse intersystem crossing (rISC) in TADF emitters is given by the Arrhenius equation K_rISC_ = A*exp*(–ΔE_ST_/K_B_T). In the early stages of the development of TADF emitters, most of the research efforts were devoted to minimizing singlet (S_1_) and triplet (T_1_) energy gap (ΔE_ST_) and thereby thermally activating rISC to achieve the delayed emission from an excited singlet state (Chiang et al., 2013; Chen et al., 2014; Peng et al., 2015; Cho et al., 2016). In general, the twisted D–A-type molecular architecture is required to minimize ΔE_ST_ by spatially isolating frontier orbitals (HOMO and LUMO) to realize TADF properties (Tao et al., 2014). However, the large twist angle between D and A not only reduces the ΔE_ST_ value but also reduces the oscillator strength of the transition (Liang et al., 2019). The temperature dependence and poor oscillator strength collectively affect the luminescence quantum yields of luminophores, which is the most important parameter in terms of real-life applications. Thus, developing an alternative design strategy is of fundamental importance (El-Sayed, 1963, 1968; Tatchen et al., 2007; Bhosale et al., 2015; Hou et al., 2018). Very recently, based on advanced computational studies, Penfold et al. and others showed that the rISC process can occur independent of temperature if there exists a second-order process such as spin–vibronic coupling between the excited singlet and triplet states in a D–A system (Etherington et al., 2016, 2017; Gibson et al., 2016; Gibson and Penfold, 2017; Penfold et al., 2018). Irrespective of the rapid research progress in TADF materials, very few molecules showing rSIC independent of temperature are reported (Hudnall et al., 2009; Mao et al., 2012; Neena et al., 2018; Salla et al., 2019).

On the other hand, coordinatively unsaturated and Lewis acid characteristics of tricoordinate born-based D–A systems have been extensively exploited for developing sensors (Hudnall et al., 2009; Galbraith and James, 2010; Jäkle, 2010), luminescence materials for non-linear optics (Yuan et al., 1990; Del Rey et al., 1998), and OLEDs (Suzuki et al., 2015; Ji et al., 2017; Mellerup and Wang, 2019). Recently, aminoborane-based molecular systems have been successfully utilized in optoelectronics (Hatakeyama et al., 2012; Wang et al., 2013; Hashimoto et al., 2014; Ayhan et al., 2016, 2018; Wang and Pei, 2016; Lien et al., 2017; Liu et al., 2017, 2019; Chen et al., 2018). We have been actively involved in developing luminescent materials by judiciously altering the molecular conformations of boron-based D–A systems (Sudhakar et al., 2013, 2017; Swamy et al., 2014; Neena and Thilagar, 2016; Kalluvettukuzhy and Thilagar, 2017). Very recently, we exploited BN/CC isosterism and topochemistry for developing highly sought-after deep-blue delayed emissive molecular systems (Neena et al., 2018).

**Figure 1:**
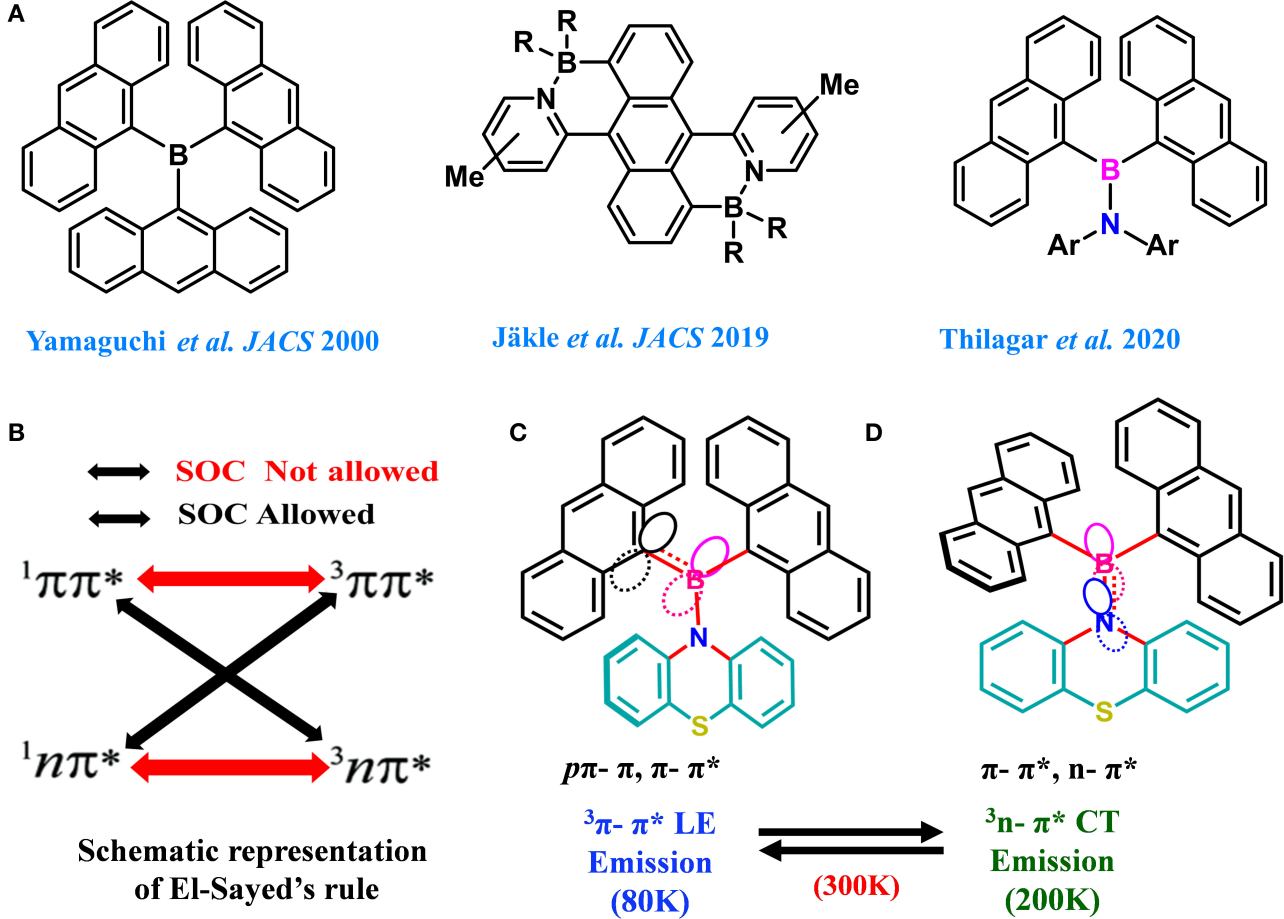
**(A)** Previously developed boron compounds and present design strategy. **(B)** Representation of El-Sayed's rule for effective spin–orbit coupling in B–N. **(C,D)** Schematic representation of the concept used for designing An_2_B-based D–A molecules with two different modes of bonding with the boron center.

In general, dimesitylboryl moiety is utilized for constructing boron-based D–A materials, because of the kinetic stability imparted by the sterically demanding mesityl group to the Lewis acidic boron center (Doty et al., 1972; Neena and Thilagar, 2016; Ji et al., 2017; Mellerup and Wang, 2019). However, the electronic coupling between boron and the attached unit is partly compromised because of steric crowding. Thus, a potential substituent could be one which maintains a good electronic coupling between the substitutes without sacrificing the overall stability of the compound. Yamaguchi et al. elegantly demonstrated the design, synthesis, and intriguing optical and anion sensing properties of a series tris(9-anthryl)borane (Figure 1A). They found that despite the large dihedral angle between the donor and acceptor units, the Lewis acid boron center had substantial π-conjugation with the anthryl units (Yamaguchi et al., 2000a,b, 2002; Wakamiya et al., 2005). Recently, Jäkle et al. explored the modification of the electronic structure of B–N-fused dipyridylanthracene and its sensitivity toward oxygen (Figure 1A) (Liu et al., 2017, 2019). Inspired by this result, we envisioned that the attachment of two anthracenyl (An) moieties to the boron center instead of sterically demanding optically innocent protecting groups (like mesityl or super mesityl) should enhance the probability of the electronic interaction between An and the boron center, leading to different optical features (Figure 1). Accordingly, we designed and synthesized di(anthrynyl)bory “An_2_B-” -based D–A systems **1** and **2** comprising An_2_B- as acceptor and donors phenothiazine and *N,N*-diphenylamine respectively, considering the following points. An attachment of two different electron donors such as “An” (π-electrons) and arylamines (nitrogen lone pair) to the electron deficient boron can be seen; consequently, different types of excited states with a distinct charge transfer character should be present. Further, the co-occurrence of excited states with different symmetries (π-π^*^ and n–π^*^) should facilitate effective spin–orbit coupling and enhance ISC and rISC processes (El-Sayed, 1963). The compact molecular structures of **1** and **2** should delicately balance the electronic coupling between the donor and acceptors to minimize ΔE_ST_; at the same time, the synergism in bonding (boron as a sigma donor and π acceptor and vice versa for C/N) between boron and the π/N moieties could make a radiative transition from the CT state efficient. Our anticipation was realized with **1** and **2** exhibiting dual TADF features with both LE and CT characterized over a broad range of temperatures from 80 to 300 K; the results are reported in this article.

## Results and Discussion

### Synthesis and Characterization

**Figure 2:**
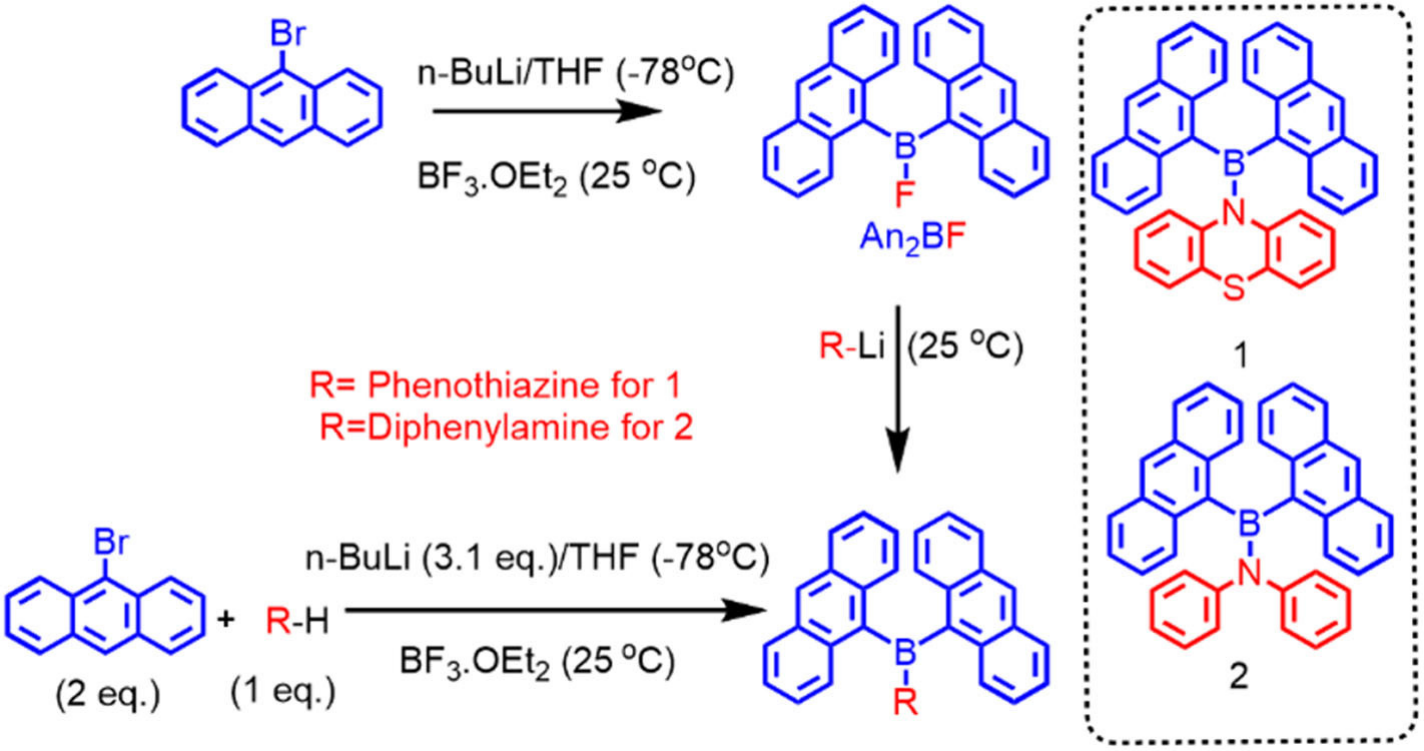
Schematic representation of the synthesis of **1** and **2**.

Precursor compound di(anthryl)boronfluoride (An_2_BF) was prepared by lithiation of 9-bromoanthracene followed by addition of 0.5 eq. BF_3_.OEt_2_. Compounds **1** and **2** were synthesized by following the procedures reported by Thilagar et al. (Sudhakar et al., 2013). Firstly, lithiation of arylamine (phenothiazine for **1** and diphenylamine for **2**) with 1 eq. of *n-*BuLi at −78°C generated an N-centered anion, which was subsequently trapped with freshly prepared An_2_BF (Pandey and Thilagar, 2020) to give **1** or **2**. Alternatively, **1** and **2** can be prepared also by one-pot synthesis as depicted in Figure 2 (see Supplementary Material file for a detailed procedure). These compounds are characterized by NMR (^1^H, ^13^C), HRMS (detailed NMR and HRMS data are given in Supplementary Figures 1–6) and single crystal x-ray diffraction analysis.

**Figure 3:**
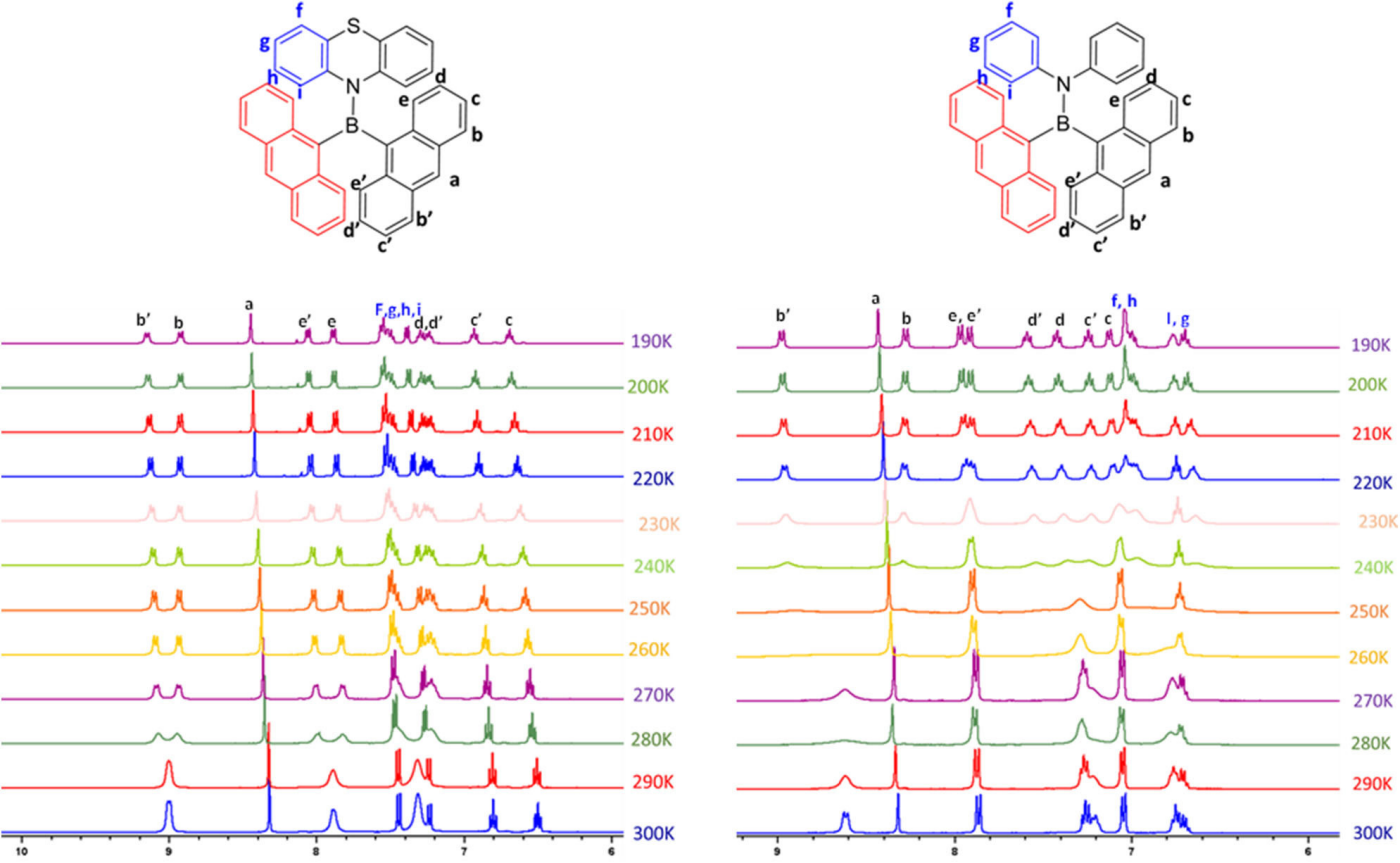
Temperature-dependent ^1^H NMR spectra of **1** (left) and **2** (right) in THF-D_8_ at a 10 K interval from 300 to 190 K.

In the case of compound **1**, out of eight ^1^H NMR peaks, three are broad whereas in the case of **2**, out of six peaks three are broad. Broad and undistinguishable peaks in the ^1^H NMR spectrum of compounds **1** and **2** reveal the presence of structural flexibility. With a view to understanding the temperature-dependent structural dynamics, the ^1^H NMR spectra of **1** and **2** were recorded at various temperatures (in THF-D8 solution, Figure 3) varying from 300 to 190 K. In Compound **1**, upon gradual cooling from 300 to 280 K, the broad signal at ~7.8 and 9.0 ppm breaks into two distinct peaks and signals from ~7.2 to 7.4 ppm get fused, which reappears as two sets of broad signals at 260 K. However, on further lowering the temperature by 220 K, signals from ~7.2 to 7.4 ppm split into three set of peaks; these spectral features remain unchanged up to 190 K. Similarly, in the case of **2**, upon gradual cooling from 300 to 270 K, the broad signal at ~9.0 ppm disappears, whereas the signal at ~6.5–7.5 ppm became broad. Upon lowering the temperature further from 270 to 250 K, a new set of signals comprising ten peaks reappear and achieve a clear and illustrious peak at 220 K. These spectral features remain constant up to 190 K. The free energy of activation (ΔG^#^) for the structural reorganization was calculated using the Gutowsky–Holm equation [ΔG^#^ = 0.00457 Tc (9.97 + log (Tc/Δδ)] (Mao et al., 2012), where Δδ = difference between the two peaks at low temperature Tc = coalescence temperature (280 and 250 K for **1** and **2** respectively). The activation–rotation energy (structural reorganization) of **1** and **2** was found to be 16.9 and 14.3 Kcal/mol, respectively. The lower ΔG^#^ of **2** suggests more structure flexibility than that of **1**. These results clearly indicate that at room temperature these molecules are in dynamic molecular motions. Thus, the electronic interactions between boron and the different functional moieties (anthracene and amine donor) were expected to show temperature-dependent optical features. As anticipated, both the compounds show temperature-dependent optical features, which are summarized vide infra. Molecular structures of these compounds were confirmed by single crystal x-ray diffraction studies (crystal data and refinement details are summarized in Supplementary Figures 7, 8, Supplementary Table 1). Compounds **1** and **2** are stable under ambient conditions toward both air and moisture.

### Molecular Structure

**Figure 4:**
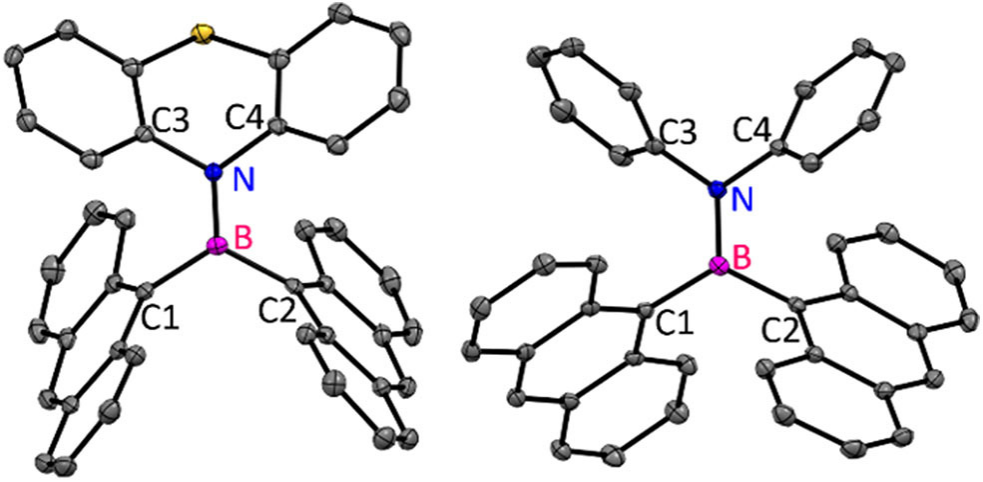
Molecular structures of **1** and **2** (ORTEP diagram with 50% probability; atom color codes: C—gray, S—yellow, N—blue, B—magenta). All the hydrogen atoms were omitted for clarity.

Compound **1** crystallized in the monoclinic crystal lattice with the *P*2_1_/n space group, whereas **2** acquired a triclinic crystal lattice and *P*-1 space group (Figure 4). In **1** and **2**, both nitrogen and boron centers adopt a trigonal planar geometry with the sum of bond angles being 360°. The B–N bond length [**1** and **2** (1.414 Å)] and dihedral angles (4.40° for **1**, 16.68° for **2**) between the borylanthracene (C1B1C2) plane and amine NC2 plane (C3N1C4) observed in **1** and **2** are shorter than the values observed for the mesityl analog (B–N; 1.430 Å, dihedral angle 23.51°) in literatures (Kalluvettukuzhy and Thilagar, 2017). Such a small dihedral angle and shorter B–N bond affirm the presence of the π-bonding interaction between the vacant p-orbital of boron and the lone pair electrons of the nitrogen atom of donor amine moieties (Kalluvettukuzhy and Thilagar, 2017). Based on these observations, one can tentatively conclude that the electronic coupling between donor bisarylamine and acceptor An_2_B- is stronger, and this interaction is expected to influence the luminescence properties of **1** and **2**. In the solid state, both the compounds show an intermolecular slipped π-π (3.371 Å in **1** and 3.344 Å for **2**) interaction with a large slip angle (58.25° in **1** and 60.09° for **2**) and CH–π interactions (2.795 Å for **1** and 2.880 Å for **2**) between anthracene units of neighboring molecules, augmenting to hold these two-neighboring anthracenes in orthogonal conformations, which significantly prevents face-to-face stacking (detailed supra-molecular interactions are given in Supplementary Figures 7, 8).

### Optical Properties

**Figure 5:**
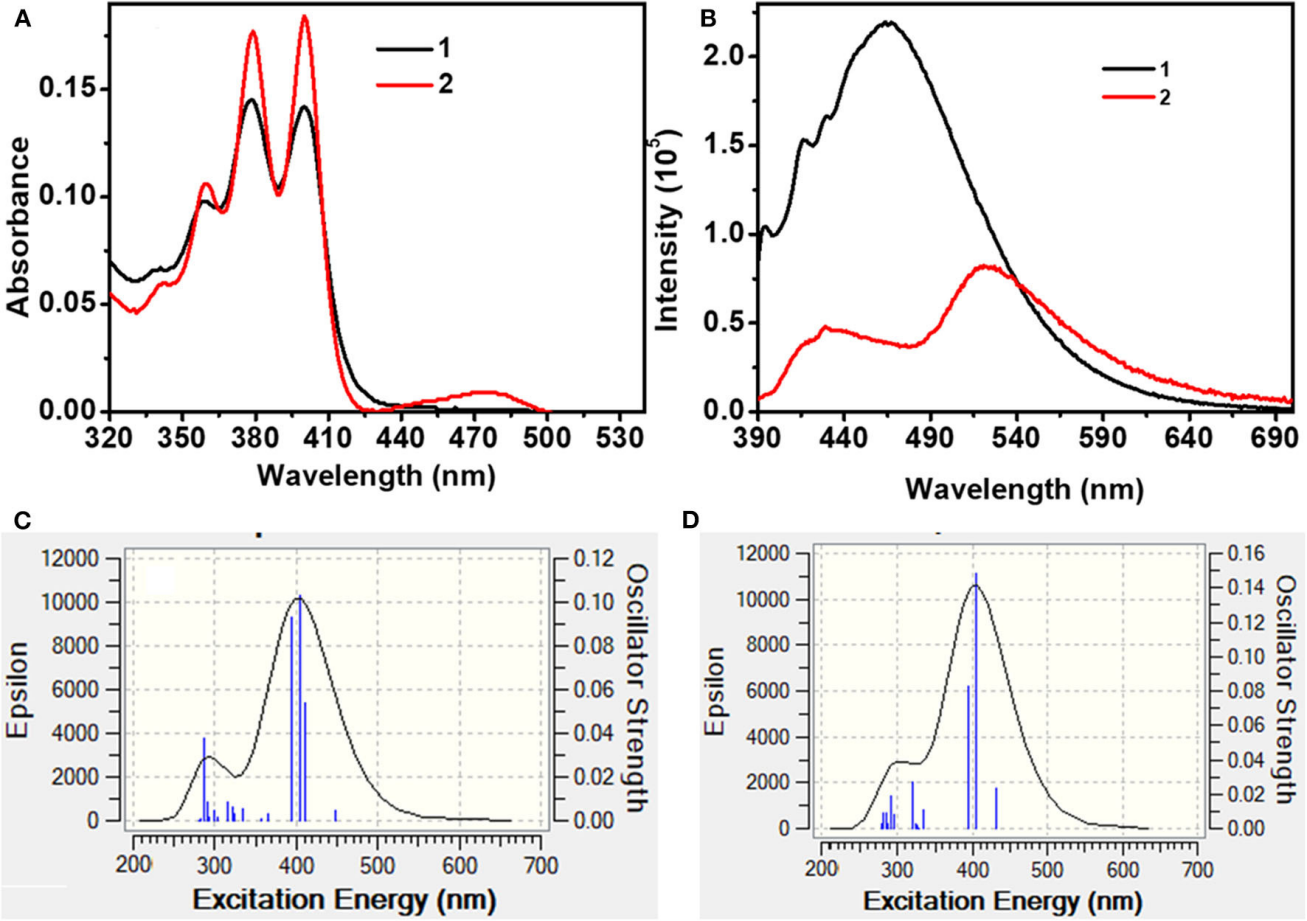
**(A)** UV-Vis and **(B)** photoluminescence spectra of **1** and **2** in toluene (Conc. 10^−5^ M; λex = 380 nm). **(C)** and **(D)** are simulated (TD-DFT) UV-Vis spectra of **1** and **2**, respectively.

In the molecularly dispersed state, both **1** and **2** show similar absorption features with distinct vibrational bands in the range 350–410 nm (Figure 5A, Table 1). However, the absorption bands are 50 nm red shifted compared to the spectra observed for simple anthracene. In addition to the structured absorption band, **2** showed a distinct structureless band at ~475 nm, which could be a charge transfer band. In contrast, **1** showed a very weak tailing on the red end of the absorption band (~425 nm). The absorption spectra of both **1** and **2** are sensitive to solvent polarity (Supplementary Figure 9). These results clearly indicate that in **1**, the electronic coupling between phenothiazine donor and An_2_B- is negligible in the ground state because of the restricted rotation around the B–N bond as a result of cyclic and rigid phenothiazine structures. However, such restrictions around B–N rotation are lifted in **2** because of a structurally flexible diphenylamine (DPA) moiety, leading to a better electronic coupling between An_2_B- and DPA moieties. To get an insight into the electronic transition, DFT and TD-DFT calculations were performed (vide supra). As depicted in **Figure 8**, Supplementary Figure 18, for **1**, the highest occupied molecular orbital (HOMO) is localized on one of the two anthracene moieties with comparatively less contribution from phenothiazine and the second anthracene moiety. In contrast, the lowest unoccupied molecular orbital (LUMO) is localized on one of the two anthracene moieties with significant contribution from the p(B) orbital and no contribution from phenothiazine and the second anthracene unit. In contrast, in **2** both the anthracene moieties and the diphenyl unit contribute to HOMO and LUMO (contribution from the p(B) orbital). The simulated UV-visible spectra of both the compounds are shown in Figures 5C,D, and the most probable transitions are listed in Supplementary Tables 7, 8. A considerably larger overlap between the frontier orbitals of **2** as compared to **1** directly corroborates with the experimentally observed intense UV-vis band for the former compared to the latter. The analysis of most probable transitions in these compounds indicates the lower energy transition HOMO → LUMO in both the compounds, the nature of CT with very low oscillator strength as compared to the higher energy transitions having an LE character and larger oscillator strength (Supplementary Tables 7, 8). These computational results support the above conclusions well. It has been well-established that electronically weakly coupled D–A systems tend to show interesting properties such as TADF (Dance et al., 2008; Tao et al., 2014). Thus, from the above results, **1** is expected show better TADF characteristics than **2**.

**Table 1:** Important optical parameters of **1** and **2**.

**Important parameters**	**1**	**2**
λ_max_, Absorption (in toluene)	376 nm	376 nm
λ_max_, Emission (in toluene)	460 nm	530 nm
Quantum yield (Φ, in toluene)^#^	0.19	0.12
Average lifetime (τ, ns, in toluene)	6.37	6.67
*k*_r_ (in toluene) (×10^6^ s^−1^)	30	18
*k*_nr_ (in toluene) (×10^6^ s^−1^)	127	132
Absolute quantum yield (Φ, solid)	0.17	0.42
Average lifetime (τ, ns, solid)	5.15	5.92
*k*_r_ (solid) (×10^6^ s^−1^)	33	71
*k*_nr_ (solid) (×10^6^ s^−1^)	161	98

# *Relative to anthracene*.

At ambient conditions, dilute solutions of **1** show a structured LE emission at ~430 nm with a CT band at ~485 nm (Figure 5B). In contrast, compound **2** showed a structureless dual-emission pattern with peak maxima at ~ 430 and 510 nm, respectively. Because of possible excited-state structural reorganization, **2** showed a structureless LE band and a red-shifted CT band compared to **1**. The PL intensity of both the compounds progressively decreased upon increasing the solvent polarity from hexane to acetonitrile (Supplementary Figure 10). In the case of **1**, in polar acetonitrile the lower-energy CT band completely disappeared; however, residual emission was observed in the higher-energy region. In contrast, under similar conditions, both the emission peaks for **2** vanished. Time-resolved emission studies on **1** and **2** in solvents with different dielectrics showed biexponential decay with short (LE) and longer (CT) lifetime components in the nanosecond range (Supplementary Table 3). The excited-state lifetime steadily decreased upon increasing the solvent polarity from hexane to methanol. These observations directly corroborate with the weaker emission observed for these compounds in polar solvents. The PL quantum yield of **1** is higher than that of **2**. Furthermore, the calculated non-radiative decay constant (k_nr_) for **2** (132 × 10^6^ s^−1^) is larger than the k_nr_ value obtained for **1** (127 × 10^6^ s^−1^), and these values are consistent with the observed PL quantum yield (Table 1).

**Figure 6:**
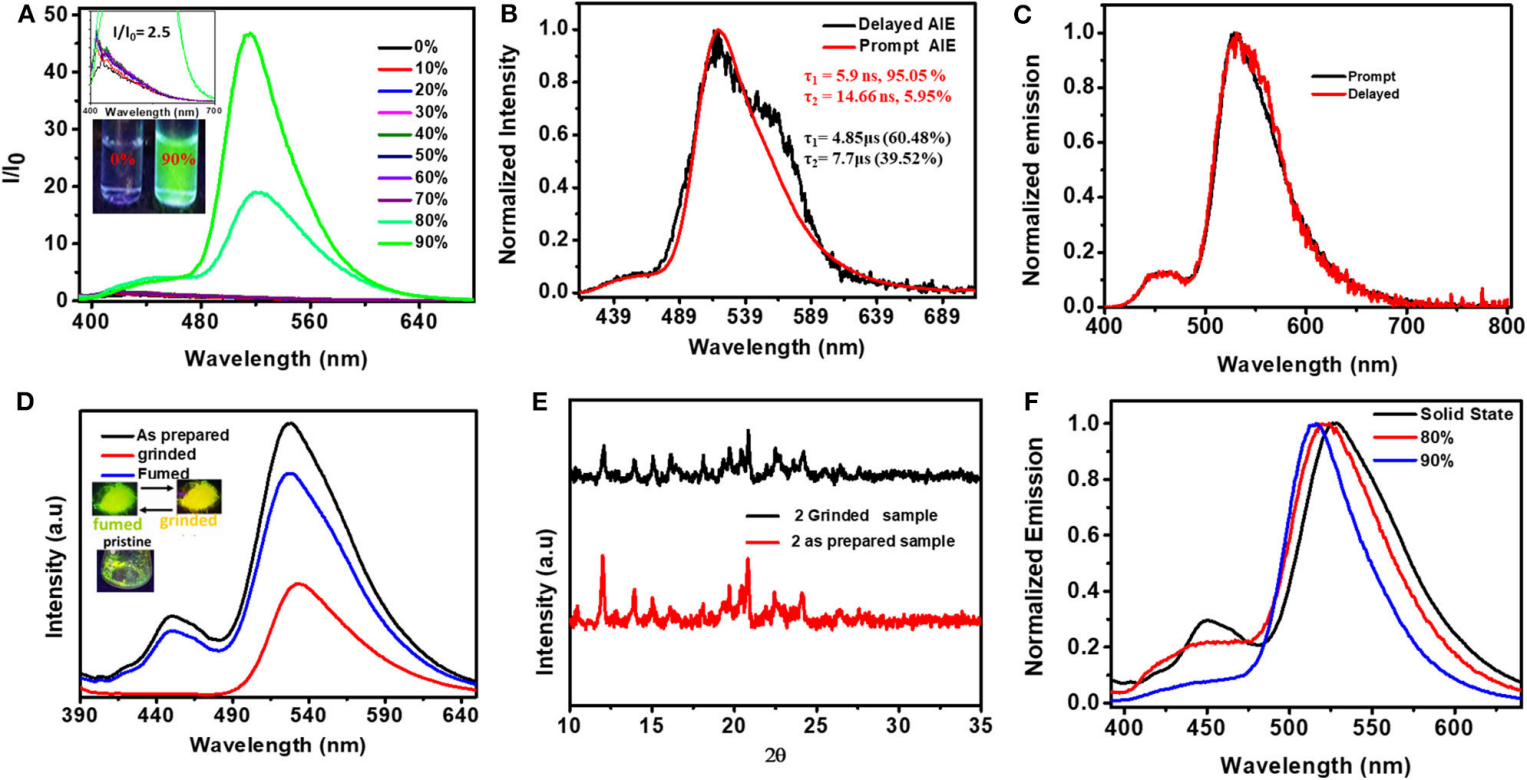
**(A)** PL spectra of **2** in THF with different fractions of water [fw (V%) (conc. 10^−5^ M, λex = 380 nm)] (inset shows the magnified emission spectra obtained for solutions with fw (V%) from 0 to 70% and the digital photograph of 2 in THF and THF/water fractions (90%), taken under UV light illumination (λex = 365 nm). **(B)** Normalized prompt and delayed PL spectra of aggregates formed in fw = 90% (Delay time 30 μs) (decay curves are given in Supplementary Figure 17). **(C)** Solid-state prompt and delayed emission spectra of **2** (λ_ex_ = 380 nm). **(D)** Mechanochromic response of **2** (inset: digital photographs of pristine solids (left), ground and DCM fumed ground samples of **2** under 365 nm UV light illumination). **(E)** PXRD pattern before and after grinding (right). **(F)** Comparative emission of **2** in solid state and aggregates formed in fw = 80 and 90%.

To understand the role of molecular flexibility in controlling the PL characteristics of these compounds, aggregation-dependent emission studies were carried out in the THF–H_2_O mixture (Figures 6A,B, Supplementary Table 4) (Luo et al., 2001; An et al., 2002). Compound **1** did not show aggregation-dependent emission. However, compound **2** showed 26-fold stronger luminescence in the aggregated state (in 90% water fraction) compared to its molecularly dispersed solutions. The lifetime of **2** in the aggregated state (in *fw* = 90%) (λ_430_, τ_av_ = 5.9 ns, λ_520_, τ_av_ = 14.44 ns) increased compared to its dilute THF solutions (λ_520_, τ_1_ = 0.87 ns, τ_2_ = 6.90 ns). Interestingly, the AIE spectral features of **2** were comparable with solid-state emission (Figure 6F). These results clearly indicate that the molecular flexibility indeed plays a role in controlling the PL characteristics of **2**. Furthermore, flexible D–A systems exhibit a mechanochromic property (Okazaki et al., 2017). When the dual emissive (greenish yellow color, 455 and 530 nm) solid of **2** was subjected to shearing forces, it showed yellow emission with a single peak at ~540 nm (Figures 6D,E). Upon exposing the yellow emissive ground powder to organic solvent vapors (such as hexane/CHCl_3_/DCM) for 3–5 min, the dual emission characteristics were restored. This process of luminescence color switching could be repeated many times and was reversible.

The prompt and delayed spectra of solids of **1** and **2** were recorded at both 300 and 80 K (Figure 6C, Supplementary Figures 13, 15). At ambient conditions, solids of **1** showed a single PL peak with a maximum at ~530 nm. This band is close to the CT emission band observed in the solution state. At 80 K, **1** showed DF characteristics similar to that observed in 300 K; in addition, it also showed a weak phosphorescence peak at ~590 nm. Interestingly, at both 300 and 80 K, the solid sample of **2** showed dual DF characteristics similar to its solution-state spectra. Further, compound **2** showed, in addition to DF peaks, a weak phosphoresce peak. The intensity of the phosphorescence peak at 80 K is stronger than that observed at 300 K. The ΔE_ST_ values for **1** (0.15 eV) and **2** (0.06) were calculated using both fluorescence and phosphorescence peaks, and the values are in the range observed for other TADF emitters reported elsewhere (Hosokai et al., 2017).

**Figure 7:**
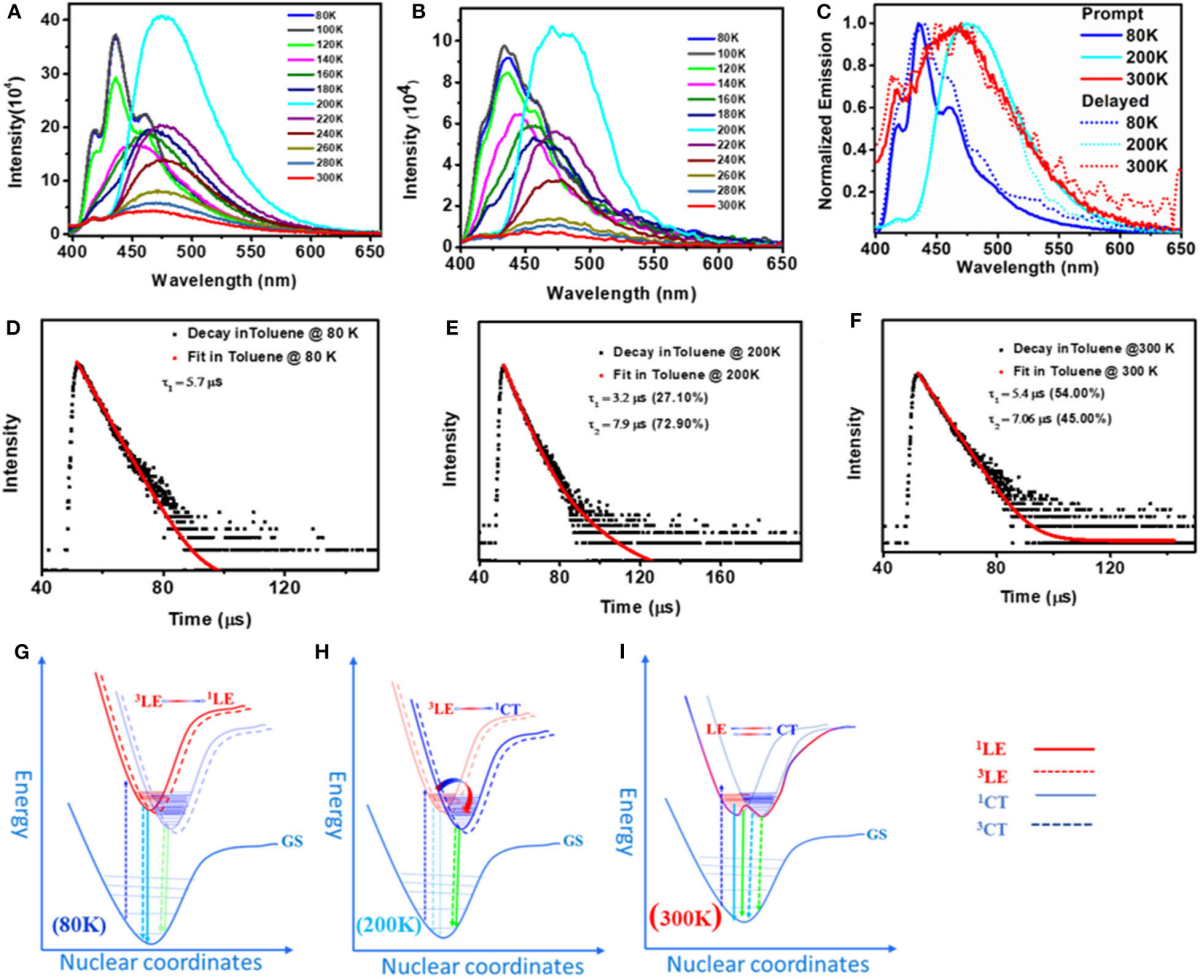
Prompt **(A)** and delayed **(B)** emission spectra of toluene solutions of **1** at 80–300 K (λex = 380 nm). **(C)** Combined delayed and prompt emission spectra of **1** at selected temperatures 80, 200, and 300 K. **(D)**, **(E)**, and **(F)** Transient decay curve with fitting for the delayed emission of **1** at 80, 200, and 300 K, respectively, measured at corresponding λ_max_. **(G)**, **(H)**, and **(I)** Proposed potential energy curves for **1** to characterize the prompt and delayed emission processes at 80, 200, and 300 K, respectively.

Apart from fascinating AIE and solid-state emission properties like mechanochromism and DF, solution-state emission at variable temperature was found to be more intriguing (Figure 7). In general, rISC is a temperature-driven process, so DF is more prominent at higher temperatures, but here we observed DF at as low as 80 K, which can also be tuned by changing the temperature. At 300 K, the prompt and delayed emission spectra of both **1** and **2** were found to overlap gently, confirming the presence of delayed fluorescence (DF). The excited-state lifetime analysis likewise indicates the formation of simultaneous two emitting species with fast [**1**; τ_1_ = 1.51 (29.59%) and τ_2_ = 8.41 ns (70.41%); **2**; τ_1_ = 1.51 (12.59%) and τ_2_ = 7.41 ns (87.41%)] and slow [**1**; τ_1_ = 5.4 (54%), τ_2_ = 7.06 (45%); **2**; τ_1_ = 5.81 (95.50%) and τ_2_ = 6.12 μs (4.50%)] components (Supplementary Table 6). It was also observed that the PL intensities of **1** and **2** were strongly quenched by oxygen indicating the involvement of a triplet excited state in the emission process (Supplementary Figure 11). To check the effect of temperature on DF characteristics of **1** and **2**, the prompt and delayed spectra of these two compounds were recorded at variable temperatures in the range 80–300 K at 20 K temperature intervals. Interestingly, irrespective of the temperature, a smooth overlap of prompt and delayed spectra of **1** and **2** was observed. However, the spectral shape and position of the peak maxima for **1** and **2** were highly sensitive to temperature at which the measurements were made. At 80 K (frozen toluene solutions), **1** and **2** showed a structured LE emission (both prompt and delayed) band and CT band at ~485 nm. When the temperature was raised from 80 to 200 K, the intensity of the LE emission decreased and the intensity of CT band at ~475 nm progressively increased and became saturated at 200 K (unfrozen toluene).

Further increase in solution temperature steadily decreased the intensity of the new band. These spectral changes ceased at 300 K, where the intensity of both the emission bands became virtually equal (Figures 7A,B). Now the pressing question which needs answer is, why do these compounds show different emission patterns at different temperatures? At 80 K, frozen toluene provides a very rigid matrix; as a result, structural reorganization is minimized and LE emission dominates. At 200 K, just above the melting temperature of toluene (178 K), the loss of matrix rigidity provides a fluidic environment for structural reorganization, leading to a molecular conformation where the CT emission dominates. At 300 K, the solvent environment is more fluidic than at 200 K; both the species are equally populated and show dual emission with equal intensity. Compound **2** shows similar but less systematic temperature-dependent spectral changes (Supplementary Figure 12).

Time-resolved excited-state decay kinetics was performed on both compounds at different temperatures. At all temperatures, both the compounds show biexponential decay with slow (microsecond) and fast (nanosecond) components (Supplementary Tables 5, 6). Further, the change in lifetime and the amplitude of the corresponding peaks directly corroborate with the change in intensity of the PL peaks observed at different temperatures (Figure 7, Supplementary Tables 5, 6). Based on steady-state and temperature-dependent time-resolved excited-state decay studies, the structured emission peak at ~435 nm is ascribed to LE emission and the slower-decaying peak is assigned to CT emission. To the best of our knowledge, simultaneous DF emission from both ^3^LE and ^3^CT was not reported in literature.

### Theoretical Studies

**Figure 8:**
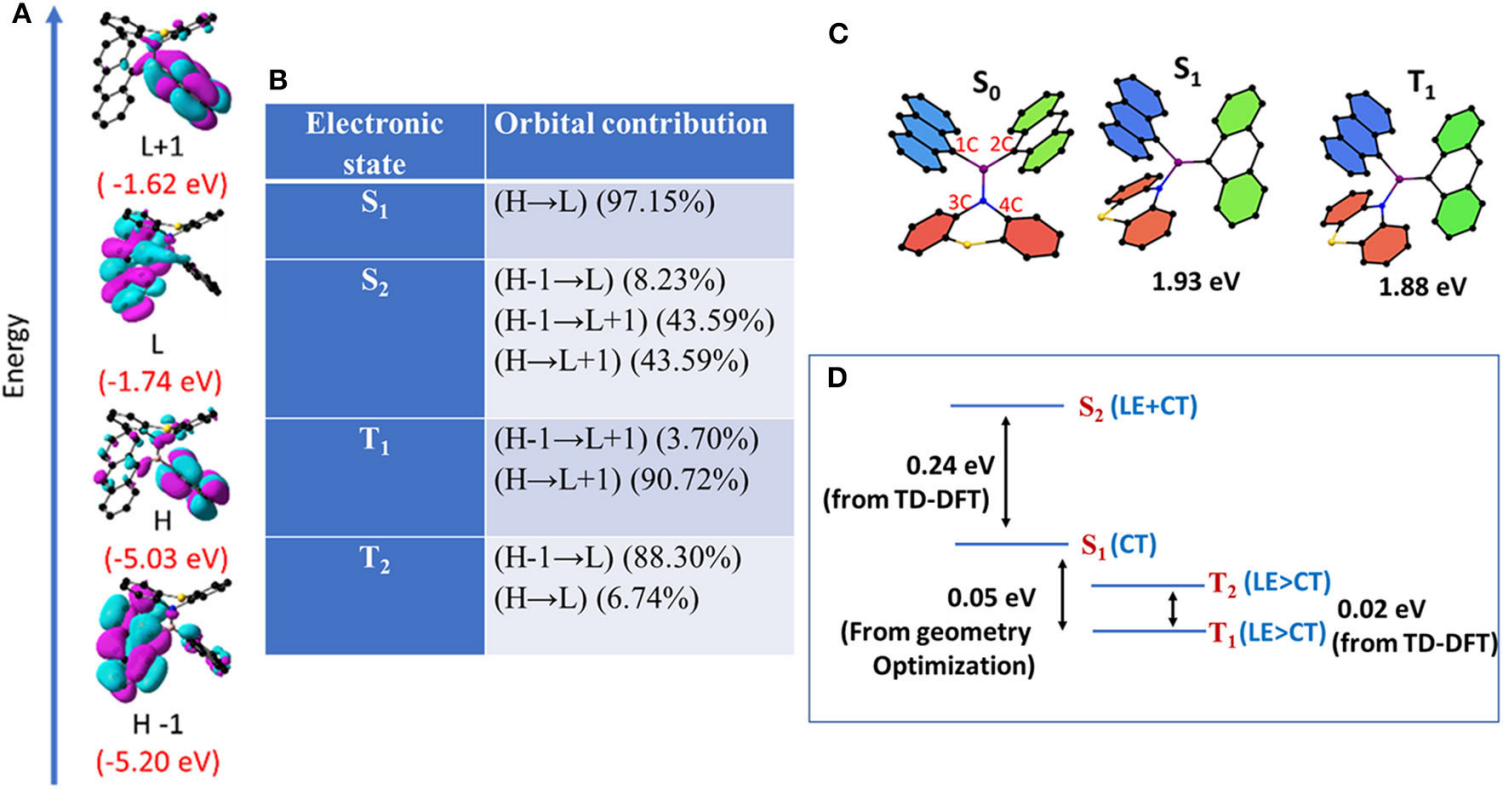
**(A)** FMOs (H = HOMO, L= LUMO) of **1** in ground state with corresponding energies. **(B)** Orbital contribution of excited states obtained from TD-DFT calculation. **(C)** Optimized geometries of **1** in S_0_, S_1_, and T_1_. **(D)** Energies and characters of the various excited states obtained from DFT and TD-DFT calculation.

With a view to understanding the optical features and electronic structure of **1** and **2**, both DFT and TD-DFT studies were carried out (Figure 8). The ground (S_0_) and excited states (S_1_, S_2_, and T_1_) geometries of **1** and **2** were also optimized. The excited state (S_1_) dipole moments are larger than the corresponding ground state in both **1** and **2**. Based on steady state, time-resolved experiments, together with computational studies, one can draw the following conclusions. Both the compounds are dual emissive; the higher energy bands may be ascribed to LE (π → π^*^ centered on anthracene moieties) and the lower-energy transitions ascribed to CT emissions [π(anthracene) → p(B)^*^, N → p(B)^*^, and π(anthracene) → π(anthracene)^*^]. Furthermore, the excited state dipole moment of **2** is much larger than that calculated for **1**. Compound **2** with flexible donor moiety undergoes molecular conformational changes required to stabilize the CT state. These conformational changes lead to the mixing of both LE and CT states to form a hybridized state (Li et al., 2014). However, such a conformational change is restricted in **1** because of molecular rigidity. Thus, the higher-energy emission peak in **1** is more of LE character and is less sensitive to solvent polarity than the peak observed for compound **2**, for which both the peaks display largely CT characteristics.

The excited-state geometries of **1** and **2** were optimized in S_1_ and T_1_ states using time-dependent density functional theory (TD-DFT). Optimization of S_2_ and T_2_ states did not converge as the calculations were computationally expensive. Energies and characters of S_2_ and T_2_ states were analyzed using the data from the vertical transitions (Noda et al., 2019). For compound **1**, in S_0_, both the anthracene units adopt a planar geometry while phenothiazine adopts a puckered geometry (Figure 8, Table 2). In contrast, the phenothiazine moiety adopts a more planar geometry and placed nearly perpendicular to the plane of both the anthryl moiety in T_1_ and S_1_ compared to the S_0_ state. To accommodate this conformational change in phenothiazine, B–N bond length is elongated and one of the two An moiety adopts a puckered geometry. Further, the bonding between boron and puckered An is shortened (Figure 8, Table 2). Energy and geometric parameters of S_2_ state were found to be very similar to S_1_. It has been demonstrated in literature that structural distortion in aromatic planar compounds, especially out-plane bending, mixes the sigma character in π-symmetry orbitals and relaxes ISC selection rules, ultimately leading to the mixing of singlet and triplet states (El-Sayed, 1968; Bhosale et al., 2015). It is clear from computational data that in compound **1**, one of the anthracene units is puckered and partially loses its π-symmetry and this structural perturbation can facilitate the out-of-plane bending of An-coupled electronic transitions (Figure 8). In the simulated IR spectra of **1** in S_1_ and T_1_, the out-of-plane bending mode of anthracene is very strong and shifted to a higher wave number compared to S_0_ (Supplementary Figure 20). These results suggest that this particular molecular vibrational mode is coupled with electronic transitions involving S_0_, S_1_, and T_1_ states (Tatchen et al., 2007).

**Table 2:** Various geometrical parameters of 1 in ground and excited states (obtained from DFT and TD-DFT calculation).

**State**	**S**_**0**_	**S**_**1**_	**T**_**1**_
PTZ twist angle (°)	44.15	22.77	18.08
An twist angle (°)	0	14.55	12.05
B-N (Å)	1.444	1.618	1.607
B-C(green) (Å)	1.611	1.476	1.490
B-C (blue) (Å)	1.602	1.613	1.605
Angle between planes B C9, C9′ (An) and N C, C′ (Ph) (°)	0.62	85.46	70.73

Further, S_1_ geometry of **1** resembles with T_1_ very closely and the D and A moieties are placed nearly orthogonally; however, they differ significantly from the S_0_ geometry where the D and A units are in planar arrangements. In contrast, in the case of **2**, the D and A moieties do not deviate much from the planarity and the geometry of S_0_, S_1_, and T_1_ resemble each other. These data further confirm the weak electronic coupling in **1** and a strong coupling in **2**. The weak coupling together with orthogonal arrangement of D and A units in **1** favors the spatial localization of frontier orbitals and reduces the exchange energy between the electronic excited states of different spin multiplicities involved in ISC/rISC events (El-Sayed, 1963, 1968; Tatchen et al., 2007; Dance et al., 2008; Tao et al., 2014; Bhosale et al., 2015; Hou et al., 2018).

The calculated (ΔE_ST_) value for optimized geometries of **1** (0.05 eV) is lower as compared to the ΔE_ST_ value calculated for **2** (ΔE_ST_ = 1.20 eV) with a large spatial overlap. Very recently, Di et al. (2017) demonstrated that ΔE_ST_ is the function of the D–A twist angle; thus, geometrical reorganization is the key driving force for ISC in D–A systems. Hence, it is reasonable to hypothesize that compound **2**, with its flexible molecular structure and free rotation around the B–N bond, can adopt a conformation in which the D–A twist angle reduces ΔE_ST_ and favors ISC/rISC events and subsequently shows DF. Based on both experimental computational results and literature precedents, we conclude that the structural features of **1** and **2** enable them to assume a dynamic conformation in which an electronic interaction between An with another An through boron results in a covalent LE state and/or interaction of An with amine through B atom results in a CT state. Thus, these compounds exhibit dual (LE and CT) delayed emissions irrespective of the temperature (Figure 7). Since TADF is a temperature-driven process, TADF molecules show DF at higher temperatures and show phosphoresce at low temperatures. Interestingly, in the present case, the dual DF characters are dependent on temperature, and it warrants explanation. Recent theoretical and experimental studies from several groups showed that the rate of rISC depends on both vibrionic coupling between ^3^CT and ^3^LE, and SOC between ^3^LE and ^1^CT (Albrecht, 1963; Etherington et al., 2016, 2017; Gibson et al., 2016; Gibson and Penfold, 2017; Penfold et al., 2018). These studies also suggest that in certain cases, such as thermally activated delayed fluorescence (TADF), the singlet–triplet crossing occurs from an adiabatically lower-lying electronic state to a higher-lying one (Gibson and Penfold, 2017). “As non-radiative transitions proceed under energy conservation, these can only be achieved if the initial state is vibrationally excited, be it thermally or kinetically” (Gibson et al., 2016; Etherington et al., 2017; Gibson and Penfold, 2017). As depicted in Figure 8 and Table 2, for **1**, S_1_ is mainly of CT character, S_2_ is of combined LE and CT character, and T_1_ and T_2_ are mainly of LE character. The very small energy differences between S_1_, T_1_, and T_2_ and their symmetry favor the intersystem crossing (and rISC) greatly. A large energy difference between S_2_ and S_1_ rules out the possibility of S_2_ to take part in the ISC or rISC processes. On the other hand, for **2** (Supplementary Figure 19), the larger energy gap between S_1_ and T_1_ may hinder the reverse intersystem crossing rate, but the combined LE and CT character of S_1_, S_2_, T_1_, and T_2_ (which relaxes spin selection rule) enables the reverse intersystem crossing. As a result, in both **1** and **2** structured luminescence peaks in the blue edge of the spectrum suggest that the first state is vibrationally excited. Further, the PL pattern of these molecules clearly indicates the presence of both CT and LE states and provides a strong platform for the vibrionic coupling between these two states. Thus, regardless of temperature, mixing of CT and LE states in **1** and **2** gives rise to a considerable rISC-mediated population transfer, leading to TADF characteristics even at low temperature.

## Conclusions

In conclusion, we reported the successful design, synthesis, and optical characteristics of a new An2B-based D–A system, in which the electronic coupling between the D and A units was controlled by the structural flexibility of the amine moiety present in them. Compound **1** with a rigid cyclic donor shows weak electronic coupling, while **2** with a flexible amine donor exhibits strong coupling. Consequently, **1** shows dual DF characteristics with strong LE and weaker CT emission bands whereas **2** shows DF with strong CT and weaker LE emission bands. Both the compounds show intriguing temperature-dependent dual DF characteristics in the range 300–80 K. Using both experimental and computational studies, it was established that the temperature-dependent dynamic molecular conformational changes in **1** and **2** led to the differential interactions between donor and acceptor units and subsequently exhibited different dual (LE and CT) DF in range of temperatures. Such structural dynamics are restricted in solid states of **1** and **2**; consequently, they exhibit temperature-dependent DF characteristics. This study also revealed that replacing mesityl in boryl acceptors with strongly optically active anthracene enhanced the probability of ISC and exhibit optical properties more promising than the former. We expect that the design strategy disclosed in this study will add a new horizon to the development of delayed emissive luminophores. These results would also attract the attentions of both experimental and computational scientist to unravel the hidden potential of this type of molecules.

## Experimental Section

### Materials and Methods

All the chemicals were purchased from commercial suppliers (Aldrich, USA; Merck, Germany; SDFCL, India) and used as received, unless otherwise mentioned. A standard Schlenk technique was used for reactions requiring inert nitrogen atmosphere THF which was dried over sodium and distilled out under nitrogen atmosphere.

400 MHz ^1^H NMR and 100 MHz ^13^C NMR spectra were recorded by a Bruker Advance 100 MHz NMR Spectrometer. Solution ^1^H NMR and ^13^C NMR spectra were referenced internally to the solvent signals. ^1^H NMR spectra were referenced to TMS (0.00 ppm) as an internal standard. Chemical shift multiplicities are reported as singlet (s), doublet (d), triplet (t), quartet (q), and multiple (m). ^13^C resonances were referenced to the CDCl_3_ signal at ~77.67 ppm. Solutions of all the compounds for spectral measurements were prepared using spectrophotometric grade solvents, microbalance (±0.1 mg precision), and standard volumetric glasswares. Quartz cuvettes with sealing screw caps were used for the solution-state spectral measurements. Electronic absorption spectra were recorded on a SHIMADZU UV-2600 spectrophotometer. The emission and excitation spectra were recorded using an Edinburgh Instruments FLS980 spectrometer. Time-gated emission spectra were recorded by an excitation source of pulsed microsecond flash lamp (μF1) with a pulse width of 1.1 μS. Single-crystal X-ray diffraction (SCXRD) studies were carried out with a Bruker SMART APEX diffractometer equipped with a 4-axis goniometer. The data were integrated using SAINT, and an empirical absorption correction was applied with SADABS. The structures were solved by direct methods and refined by full matrix least squares on F^2^ using SHELXTL software.

Density functional theory (DFT/TD-DFT) calculations were done using B3LYP functional with a 6-31G(d,p) basis set as incorporated in the Gaussian 09 package for all the atoms, mixing the exact Hartree–Fock-type exchange with Becke's exchange functional and that proposed by Lee–Yang–Parr for the correlation contribution (Frisch et al., 2009). The molecular structures obtained from SCXRD measurements were taken as the input for the calculations. The optimized structures and the frontier molecular orbitals (FMOs) were viewed using GaussView 5.0 (Dennington et al., 2009).

### Synthesis and Characterization

#### Synthesis of 1

9-Bromoanthracene (2 g, 7.84 mmol) and phenothiazine (0.78 g, 3.95 mmol) were taken in THF (100 mL) under nitrogen atmosphere, and n-BuLi (7.95 mL of a 1.6-M solution in hexanes, 12.93 mmol) was added at −78°C under stirring conditions. The reaction mixture was stirred for 1 h at the same temperature, and BF_3_·OEt_2_ (0.4 mL, 3.74 mmol) was added. The resultant reaction mixture was allowed to warm to room temperature, and stirring was continued for an additional 10 h. All the volatiles were removed under reduced pressure, and the crude product was extracted with ethyl acetate. The combined ethyl acetate extracts were stored over anhydrous Na_2_SO_4_, and the solvent was removed under a reduced pressure which gave a crude product. An analytically pure compound was obtained after column chromatography over alumina using 10% EtOAc in hexane as eluent. Yellow solid, yield: 590 mg; 27%. ^1^H NMR (400 MHz, CDCl_3_, 25°C): δ (ppm) 8.91–8.93 (broad, 4 H), 8.28 (s, 2H), 7.33–7.35 (m, 10 H), 7.20 (d, *J* = 8.0 Hz, 4H), 6.79 (t, *J* = 8.0 Hz 2H), 6.49 (t, *J* = 8.0 Hz, 2H). ^13^C NMR (100 MHz, CDCl_3_, 25°C): δ (ppm) 143.67, 131.07, 130.65, 129.88, 129.30, 128.78, 127.33, 126.34, 125.44, 125.17, 124.50. HRMS (Q-TOF): m/z calculated for [C_40_H_26_BNSNa]^+^ 586.1773, found [M+Na]^+^ 586.1777.

#### Synthesis of 2

Compound **2** was synthesized following the procedure used for the synthesis of **1**. Reagents used, quantities involved, and the characterization data as follows. 9-Bromoanthracene (2 g, 7.84 mmol), diphenylamine (0.67 g, 3.95 mmol), n-BuLi (7.95 mL of a 1.6-M solution in hexanes, 12.93 mmol), and BF_3_·OEt_2_ (0.4 mL, 3.74 mmol). Greenish yellow solid, yield: 510 mg; 25%. ^1^H NMR (400 MHz, CDCl_3_, 25°C): δ (ppm) 8.54 (broad, 4 H), 8.30 (s, 2H), 7.86–7.89 (m, 4 H), 7.26–7.30 (m, 8H), 7.00–7.02 (broad, 4H), 6.73–6.76 (broad, 6H). ^13^C NMR (100 MHz, CDCl_3_, 25°C): δ (ppm) 149.47, 131.58, 129.60, 129.25, 129.07, 128.23, 127.50, 126.49, 125.83, 125.44, 125.94. HRMS (Q-TOF): m/z calculated for C_40_H_28_BN 533.2315 [M^+^]; found 533.2321.

## Data Availability Statement

Publicly available datasets were analyzed in this study. This data can be found here: (https://www.ccdc.cam.ac.uk/structures/) CCDC 1954827 and CCDC 1952521.

## Author Contributions

UP did compound synthesis and PL characterization. UP and RN contributed to DFT calculations and data analysis. PT organized the entire project. All authors commented on the manuscript.

## Conflict of Interest

The authors declare that the research was conducted in the absence of any commercial or financial relationships that could be construed as a potential conflict of interest.

## References

[B1] Albrecht A. C. (1963). Vibronic—spin-orbit perturbations and the assignment of the lowest triplet state of benzene. J. Chem. Phys. 38, 354–365. 10.1063/1.1733665

[B2] An B.-K. Kwon S.-K. Jung S.-D. Park S. Y. (2002). Enhanced emission and its switching in fluorescent organic nanoparticles. J. Am. Chem. Soc. 124, 14410–14415. 10.1021/ja0269082 12452716

[B3] Ayhan O. Eckert T. Plamper F. A. Helten H. (2016). Poly(iminoborane)s: an elusive class of main-group polymers? Angew. Chem. Int. Ed. 55, 13321–13325. 10.1002/anie.201607131 27651296

[B4] Ayhan O. Riensch N. A. Glasmacher C. Helten H. (2018). Cyclolinear oligo- and poly(iminoborane)s: the missing link in inorganic main-group macromolecular chemistry. Chemistry 24, 5883–5894. 10.1002/chem.201705913 29377367

[B5] Bhosale R. S. Al Kobaisi M. Bhosale S. V. Bhargava S. Bhosale S. V. (2015). Flower-like supramolecular self-assembly of phosphonic acid appended naphthalene diimide and melamine. Sci. Rep. 5:14609. 10.1038/srep14609 26416382 PMC4586721

[B6] Chen D. G. Lin T. C. Chen C. L. Chen Y. T. Chen Y. A. Lee G. H.. (2018). Optically triggered planarization of boryl-substituted phenoxazine: another horizon of TADF molecules and high-performance OLEDs. ACS Appl. Mater. Interfaces 10, 12886–12896. 10.1021/acsami.8b00053 29582654

[B7] Chen L. Jiang Y. Nie H. Hu R. Kwok H. S. Huang F.. (2014). Rational design of aggregation-induced emission luminogen with weak electron donor–acceptor interaction to achieve highly efficient undoped bilayer OLEDs. ACS Appl. Mater. Interfaces 6, 17215–17225. 10.1021/am505036a 25254940

[B8] Chiang C.-J. Kimyonok A. Etherington M. K. Griffiths G. C. Jankus V. Turksoy F. (2013). Ultrahigh Efficiency Fluorescent Single and Bi-Layer Organic Light Emitting Diodes: The Key Role of Triplet Fusion. Adv. Funct. Mater 23, 739–746. 10.1002/adfm.201201750

[B9] Cho Y. J. Zhang Y. Yu H. Aziz H. (2016). The Root Causes of the Limited Stability of Solution-Coated Small-Molecule Organic Light-Emitting Devices: Faster Host Aggregation by Exciton–Polaron Interactions. Adv. Funct. Mater 26, 8662–8669. 10.1002/adfm.201603542

[B10] Dance Z. E. X. Mickley S. M. Wilson T. M. Ricks A. B. Scott A. M. Ratner M. A.. (2008). Intersystem crossing mediated by photoinduced intramolecular charge transfer: julolidine–anthracene molecules with perpendicular π systems. J. Phys. Chem. A 112, 4194–4201. 10.1021/jp800561g 18386857

[B11] Del Rey B. Keller U. Torres T. Rojo G. Agulló-López F. Nonell S. (1998). Synthesis and nonlinear optical, photophysical, and electrochemical properties of subphthalocyanines. J. Am. Chem. Soc. 120, 12808–12817. 10.1021/ja980508q

[B12] Dennington R. Keith T.A. Millam J.M. (2009). GaussView 5.0.8. Shawnee, KS: Semichem Inc.

[B13] Di D. Romanov A. S. Yang L. Richter J. M. Rivett J. P. H. Jones S.. (2017). High-performance light-emitting diodes based on carbene-metal-amides. Science 356, 159–163. 10.1126/science.aah4345 28360136

[B14] Doty J. C. Babb B. Grisdale P. J. Glogowski M. Williams J. L. R. (1972). Boron photochemistry: IX. Synthesis and fluorescent properties of dimesityl-phenylboranes. J. Organomet. Chem. 38, 229–236. 10.1016/S0022-328X(00)83321-5

[B15] El-Sayed M. A. (1963). Spin—orbit coupling and the radiationless processes in nitrogen heterocyclics. J. Chem. Phys. 38, 2834–2838. 10.1063/1.1733610

[B16] El-Sayed M. A. (1968). Triplet state. Its radiative and nonradiative properties. Acc. Chem. Res. 1, 8–16. 10.1021/ar50001a002

[B17] Etherington M. K. Franchello F. Gibson J. Northey T. Santos J. Ward J. S.. (2017). Regio- and conformational isomerization critical to design of efficient thermally-activated delayed fluorescence emitters. Nat. Commun. 8:14987. 10.1038/ncomms14987 28406153 PMC5399286

[B18] Etherington M. K. Gibson J. Higginbotham H. F. Penfold T. J. Monkman A. P. (2016). Revealing the spin–vibronic coupling mechanism of thermally activated delayed fluorescence. Nat. Commun. 7:13680. 10.1038/ncomms13680 27901046 PMC5141373

[B19] Frisch M. J. Trucks G. W. Schlegel H. B. Scuseria G. E. Robb M. A. Cheeseman J. R. (2009). Gaussian09. Revision A.02. Wallingford, CT: Gaussian, Inc.

[B20] Galbraith E. James T. D. (2010). Boron based anion receptors as sensors. Chem. Soc. Rev. 39, 3831–3842. 10.1039/b926165f 20820463

[B21] Gibson J. Monkman A. P. Penfold T. J. (2016). The importance of vibronic coupling for efficient reverse intersystem crossing in thermally activated delayed fluorescence molecules. Chemphyschem 17, 2956–2961. 10.1002/cphc.201600662 27338655 PMC5096030

[B22] Gibson J. Penfold T. J. (2017). Nonadiabatic coupling reduces the activation energy in thermally activated delayed fluorescence. Phys. Chem. Chem. Phys. 19, 8428–8434. 10.1039/C7CP00719A 28286891

[B23] Hashimoto S. Ikuta T. Shiren K. Nakatsuka S. Ni J. Nakamura M. (2014). Triplet-energy control of polycyclic aromatic hydrocarbons by BN replacement: development of ambipolar host materials for phosphorescent organic light-emitting diodes. Chem. Mater. 26, 6265–6271. 10.1021/cm503102d

[B24] Hatakeyama T. Hashimoto S. Oba T. Nakamura M. (2012). Azaboradibenzo[6]helicene: carrier inversion induced by helical homochirality. J. Am. Chem. Soc. 134, 19600–19603. 10.1021/ja310372f 23167918

[B25] Hosokai T. Matsuzaki H. Nakanotani H. Tokumaru K. Tsutsui T. Furube A.. (2017). Evidence and mechanism of efficient thermally activated delayed fluorescence promoted by delocalized excited states. Sci. Adv. 3:e1603282. 10.1126/sciadv.1603282 28508081 PMC5425233

[B26] Hou Y. Biskup T. Rein S. Wang Z. Bussotti L. Russo N. (2018). Spin–orbit charge recombination intersystem crossing in phenothiazine–anthracene compact dyads: effect of molecular conformation on electronic coupling, electronic transitions, and electron spin polarizations of the triplet states. J. Phys. Chem. C 122, 27850–27865. 10.1021/acs.jpcc.8b08965

[B27] Hudnall T. W. Chiu C.-W. Gabbaï F. P. (2009). Fluoride ion recognition by chelating and cationic boranes. Acc. Chem. Res. 42, 388–397. 10.1021/ar8001816 19140747

[B28] Jäkle F. (2010). Advances in the synthesis of organoborane polymers for optical, electronic, and sensory applications. Chem. Rev. 110, 3985–4022. 10.1021/cr100026f 20536123

[B29] Ji L. Griesbeck S. Marder T. B. (2017). Recent developments in and perspectives on three-coordinate boron materials: a bright future. Chem. Sci. 8, 846–863. 10.1039/C6SC04245G 28572897 PMC5452272

[B30] Kalluvettukuzhy N. K. Thilagar P. (2017). Bistable polyaromatic aminoboranes: bright solid state emission and mechanochromism. Organometallics 36, 2692–2701. 10.1021/acs.organomet.7b00332

[B31] Kim S. Bae H. J. Park S. Kim W. Kim J. Kim J. S.. (2018). Degradation of blue-phosphorescent organic light-emitting devices involves exciton-induced generation of polaron pair within emitting layers. Nat. Commun. 9:1211. 10.1038/s41467-018-03602-4 29572485 PMC5865184

[B32] Li W. Pan Y. Yao L. Liu H. Zhang S. Wang C. (2014). A hybridized local and charge-transfer excited state for highly efficient fluorescent OLEDs: molecular design, spectral character, and full exciton utilization. Adv. Optic. Mater. 2, 892–901. 10.1002/adom.201400154

[B33] Liang X. Tu Z. L. Zheng Y. X. (2019). Thermally activated delayed fluorescence materials: towards realization of high efficiency through strategic small molecular design. Chemistry 25, 5623–5642. 10.1002/chem.201805952 30648301

[B34] Lien Y. J. Lin T. C. Yang C. C. Chiang Y. C. Chang C. H. Liu S. H.. (2017). First N-borylated emitters displaying highly efficient thermally activated delayed fluorescence and high-performance OLEDs. ACS Appl. Mater. Interfaces 9, 27090–27101. 10.1021/acsami.7b08258 28731681

[B35] Liu K. Lalancette R. A. Jäkle F. (2017). B–N Lewis pair functionalization of anthracene: structural dynamics, optoelectronic properties, and O_2_ sensitization. J. Am. Chem. Soc. 139, 18170–18173. 10.1021/jacs.7b11062 29185739

[B36] Liu K. Lalancette R. A. Jakle F. (2019). Tuning the structure and electronic properties of B-N fused dipyridylanthracene and implications on the self-sensitized reactivity with singlet oxygen. J. Am. Chem. Soc. 141, 7453–7462. 10.1021/jacs.9b01958 30998336

[B37] Luo J. Xie Z. Lam J. W. Y. Cheng L. Chen H. Qiu C. (2001). Aggregation-induced emission of 1-methyl-1,2,3,4,5-pentaphenylsilole. Chem. Commun. 381, 1740–1741. 10.1039/b105159h 12240292

[B38] Mao M. Ren M.-G. Song Q.-H. (2012). Thermodynamics and conformations in the formation of excited states and their interconversions for twisted donor-substituted tridurylboranes. Chemistry 18, 15512–15522. 10.1002/chem.201201719 23032965

[B39] Mellerup S. K. Wang S. (2019). Boron-based stimuli responsive materials. Chem. Soc. Rev. 48, 3537–3549. 10.1039/C9CS00153K 31070642

[B40] Neena K. K. Sudhakar P. Thilagar P. (2018). Catalyst- and template-free ultrafast visible-light-triggered dimerization of vinylpyridine-functionalized tetraarylaminoborane: intriguing deep-blue delayed fluorescence. Angew. Chem. Int. Ed. 57, 16806–16810. 10.1002/anie.201811353 30345594

[B41] Neena K. K. Thilagar P. (2016). Replacing the non-polarized C=C bond with an isoelectronic polarized B–N unit for the design and development of smart materials. J. Mater. Chem. C 4, 11465–11473. 10.1039/C6TC04470K

[B42] Noda H. Chen X. K. Nakanotani H. Hosokai T. Miyajima M. Notsuka N.. (2019). Critical role of intermediate electronic states for spin-flip processes in charge-transfer-type organic molecules with multiple donors and acceptors. Nat. Mater. 18, 1084–1090. 10.1038/s41563-019-0465-6 31477903

[B43] Noda H. Nakanotani H. Adachi C. (2018). Excited state engineering for efficient reverse intersystem crossing. Sci. Adv. 4:6910. 10.1126/sciadv.aao6910 29942856 PMC6014720

[B44] Northey T. Keane T. Eng J. Penfold T. J. (2019). Understanding the potential for efficient triplet harvesting with hot excitons. Faraday Discuss 216, 395–413. 10.1039/C8FD00174J 31012872

[B45] Obolda A. Ai X. Zhang M. Li F. (2016). Up to 100% formation ratio of doublet exciton in deep-red organic light-emitting diodes based on neutral pi-radical. ACS Appl. Mater. Interfaces 8, 35472–35478. 10.1021/acsami.6b12338 27933759

[B46] Okazaki M. Takeda Y. Data P. Pander P. Higginbotham H. Monkman A. P.. (2017). Thermally activated delayed fluorescent phenothiazine-dibenzo[a,j]phenazine-phenothiazine triads exhibiting tricolor-changing mechanochromic luminescence. Chem. Sci. 8, 2677–2686. 10.1039/C6SC04863C 28553504 PMC5433494

[B47] Pandey U. P. Thilagar P. (2020). External stimuli responsive bis(anthryl)borylaniline AIEgens for viscosity and temperature sensing: the game of molecular flexibility. Adv. Opt. Mater. 8:1902145 10.1002/adom.201902145

[B48] Penfold T. J. Gindensperger E. Daniel C. Marian C. M. (2018). Spin-vibronic mechanism for intersystem crossing. Chem. Rev. 118, 6975–7025. 10.1021/acs.chemrev.7b00617 29558159

[B49] Peng Q. Obolda A. Zhang M. Li F. (2015). Organic light-emitting diodes using a neutral pi radical as emitter: the emission from a doublet. Angew. Chem. Int. Ed. 54, 7091–7095. 10.1002/anie.201500242 25916621

[B50] Pu Y.-J. Satake R. Koyama Y. Otomo T. Hayashi R. Haruta N. (2019). Absence of delayed fluorescence and triplet–triplet annihilation in organic light emitting diodes with spatially orthogonal bianthracenes. J. Mater. Chem. C 7, 2541–2547. 10.1039/C8TC05817B

[B51] Salla C. A. M. Farias G. Rouzières M. Dechambenoit P. Durola F. Bock H.. (2019). Persistent solid-state phosphorescence and delayed fluorescence at room temperature by a twisted hydrocarbon. Angew. Chem. Int. Ed. 58, 6982–6986. 10.1002/anie.201901672 30908833

[B52] Sudhakar P. Mukherjee S. Thilagar P. (2013). Revisiting borylanilines: unique solid-state structures and insight into photophysical properties. Organometallics 32, 3129–3133. 10.1021/om301197f

[B53] Sudhakar P. Neena K. K. Thilagar P. (2017). H-Bond assisted mechanoluminescence of borylated aryl amines: tunable emission and polymorphism. J. Mater. Chem. C 5, 6537–6546. 10.1039/C7TC01676J

[B54] Suzuki K. Kubo S. Shizu K. Fukushima T. Wakamiya A. Murata Y.. (2015). Triarylboron-based fluorescent organic light-emitting diodes with external quantum efficiencies exceeding 20%. Angew. Chem. Int. Ed. 54, 15231–15235. 10.1002/anie.201508270 26563845

[B55] Swamy P. C. A. Mukherjee S. Thilagar P. (2014). Dual binding site assisted chromogenic and fluorogenic recognition and discrimination of fluoride and cyanide by a peripherally borylated metalloporphyrin: overcoming anion interference in organoboron based sensors. Anal. Chem. 86, 3616–3624. 10.1021/ac500230p 24571811

[B56] Tao Y. Yuan K. Chen T. Xu P. Li H. Chen R.. (2014). Thermally activated delayed fluorescence materials towards the breakthrough of organoelectronics. Adv. Mater. 26, 7931–7958. 10.1002/adma.201402532 25230116

[B57] Tatchen J. Gilka N. Marian C. M. (2007). Intersystem crossing driven by vibronic spin–orbit coupling: a case study on psoralen. Phys. Chem. Chem. Phys. 9, 5209–5221. 10.1039/b706410a 19459284

[B58] Uoyama H. Goushi K. Shizu K. Nomura H. Adachi C. (2012). Highly efficient organic light-emitting diodes from delayed fluorescence. Nature 492, 234–238. 10.1038/nature11687 23235877

[B59] Wakamiya A. Ide T. Yamaguchi S. (2005). Toward π-conjugated molecule bundles: synthesis of a series of B,B′,B″-trianthryl-N,N′,N″-triarylborazines and the bundle effects on their properties. J. Am. Chem. Soc. 127, 14859–14866. 10.1021/ja0537171 16231940

[B60] Wang J.-Y. Pei J. (2016). BN-embedded aromatics for optoelectronic applications. Chin. Chem. Lett. 27, 1139–1146. 10.1016/j.cclet.2016.06.014

[B61] Wang X.-Y. Lin H.-R. Lei T. Yang D.-C. Zhuang F.-D. Wang J.-Y.. (2013). Azaborine compounds for organic field-effect transistors: efficient synthesis, remarkable stability, and BN dipole interactions. Angew. Chem. Int. Ed. 52, 3117–3120. 10.1002/anie.201209706 23400958

[B62] Yamaguchi S. Akiyama S. Tamao K. (2000a). Tri-9-anthrylborane and its derivatives: new boron-containing π-electron systems with divergently extended π-conjugation through boron. J. Am. Chem. Soc. 122, 6335–6336. 10.1021/ja994522u

[B63] Yamaguchi S. Akiyama S. Tamao K. (2002). The coordination number–photophysical properties relationship of trianthrylphosphorus compounds: doubly locked fluorescence of anthryl groups. J. Organomet. Chem. 646, 277–281. 10.1016/S0022-328X(01)01391-2

[B64] Yamaguchi S. Shirasaka T. Tamao K. (2000b). Tridurylboranes extended by three arylethynyl groups as a new family of boron-based π-electron systems. Org. Lett. 2, 4129–4132. 10.1021/ol006660q 11150181

[B65] Yang Z. Mao Z. Xie Z. Zhang Y. Liu S. Zhao J.. (2017). Recent advances in organic thermally activated delayed fluorescence materials. Chem. Soc. Rev. 46, 915–1016. 10.1039/C6CS00368K 28117864

[B66] Ye C. Gray V. Martensson J. Borjesson K. (2019). Annihilation versus excimer formation by the triplet pair in triplet-triplet annihilation photon upconversion. J. Am. Chem. Soc. 141, 9578–9584. 10.1021/jacs.9b02302 31131601 PMC6608582

[B67] Yuan Z. Taylor N. J. Marder T. B. Williams I. D. Kurtz S. K. Cheng L.-T. (1990). Three coordinate phosphorus and boron as π-donor and π-acceptor moieties respectively, in conjugated organic molecules for nonlinear optics: crystal and molecular structures of E-Ph-CH=CH-B(mes)2, E-4-MeO-C6H4-CH=CH-B(mes)2, and E-Ph 2P-CH=CH-B(mes)2 [mes = 2,4,6-Me3C 6H2]. J. Chem. Soc. Chem. Comm. 1489–1492. 10.1039/C39900001489

