# Supplementary material for: Design, Synthesis, and Temperature-Driven Molecular Conformation-Dependent Delayed Fluorescence Characteristics of Dianthrylboron-Based Donor–Acceptor Systems
Source: Front Chem. 2020 Oct 9;8:541331. doi: 10.3389/fchem.2020.541331 (PMC7581868; doi:10.3389/fchem.2020.541331)
Supplement: Supplementary file 1 [file Table_1.DOCX]

**SUPPORTING INFORMATION**

**Design, Synthesis and Temperature Driven Molecular Conformation Dependent Delayed Fluorescence Characteristics of Dianthrylboron Based Donor-Acceptor Systems**

Umesh Pratap Pandey, Rajendra Prasad Nandi and Pakkirisamy Thilagar*

Department of Inorganic and Physical Chemistry, Indian Institute of Science, Bangalore 560012, INDIA

thilagar@iisc.ac.in

**Table of Contents**

**Figure S1:** ^1^H NMR spectrum of compound **1.**

**Figure S2: ^13^**C NMR spectrum of compound **1.**

**Figure S3:** ^1^H NMR spectrum of compound **2.**

**Figure S4:** ^13^C NMR spectrum of compound **2.**

**Figure S5:** HRMS of compound **1.**

**Figure S6:** HRMS of compound **2.**

**Figure S7:** (A) Crystal structure of **1** depicting intermolecular (C-H....π), π....π Interactions (red dotted lines) and slip angle between two anthryl planes of different molecules. (B) The crystallographic axis along the picture is given.

**Figure S8:** (A) Crystal structure of **2** depicting intermolecular (C-H....π), π....π Interactions (red dotted lines) and slip angle between two anthryl planes of different molecules. (B) The crystallographic axis along the picture is given.

**Table S1:** Single crystal structure refinement data of **1** and **2.**

**Figure S9:** UV-Vis spectra of **1** (A) and **2** (B) in solvent of different polarity (Conc. 10^-5^M).

**Table S2**: Absorption maxima of individual vibrational bands (λ_max_) and corresponding ε for **1** and **2**

**Figure S10:** Photoluminescence spectra of **1** (A) and **2** (B) in solvent of different polarity (Conc. 10^-5^M; λ_ex_ = 380 nm).

**Table S3:** time resolved decay kinetics data of **1** (Left) and **2** (Right).

**Table S4:** Excited state lifetime of **2** in THF and aggregates in THF/Water mixture (10:90 %).

**Table S5:** Temperature dependent DF lifetime of **1** and **2.**

**Table S6:** Temperature dependent TCSPC lifetime of **1** and **2.**

**Figure S11:** Effect of O_2_ on DF of in **1** and **2.**

**Figure S12:** Prompt (A) and delayed (B) spectra of **2** at 80, 200K, and 300K and 5X zoom image of 200K, and 300K emission. (λ_ex_ = 380 nm; (Delay time 30 μs)) Excited state decay kinetics fitting for the DF of solution of **2** at 80K (Emission = 430 nm) (C) and 300K (Emission =430 nm (D) and 530 nm (E)) in toluene.

**Table S7**: Frontier molecular orbitals of **1** and calculated possible electronic transitions at B3LYP/631G (H = HOMO; L = LUMO).

**Table S8:** Frontier molecular orbitals of **2** and calculated possible electronic transitions at B3LYP/631G (H = HOMO; L = LUMO).

**Figure S13:** Frontier molecular orbitals of **1** and **2 (**A) and (B) respectively in ground state (left) and first excited state (right).

**Figure S14:** DFT Calculated IR spectra of **1** (A) and **2** (B) in S0, S1 and T1 states.

**Figure S15:** The prompt and delayed fluorescence of solids of **1** at 300 K.

**Figure S16:** Excited state decay kinetics fitting for the delayed DF of solids of **1** and **2** at 300 K.

**Figure S17:** The prompt and delayed fluorescence of solids of **1** and **2** at 80 K**.**

**Figure S18:** Excited state decay kinetics fitting for the delayed DF of solids of **1** and **2** at 80 K.

**Figure S19.** Excited state decay kinetics fitting for the prompt (left) and DF (right) of aggregates of **2**.

**Table S9:** Cartesian coordinates of optimized structures in S_0_ for **1,** calculated using Gaussian 09 at the B3LYP/6-31G (d,p) level of theory.

**Table S10:** Cartesian coordinates of optimized structures in S_1_ for**1** calculated using Gaussian 09 at the B3LYP/6-31G (d,p) level of theory.

**Table S11:** Cartesian coordinates of optimized structures in T_1_ for **1** calculated using Gaussian 09 at the B3LYP/6-31G (d,p) level of theory

**Table S12:** Cartesian coordinates of optimized structures in S_0_ for **2** calculated using Gaussian 09 at the B3LYP/6-31G (d, p) level of theory.

**Table S13:** Cartesian coordinates of optimized structures in S_1_ for **2** calculated using Gaussian 09 at the B3LYP/6-31G (d, p) level of theory.

**Table S14:** Cartesian coordinates of optimized structures in T_1_ for **2** calculated using Gaussian 09 at the B3LYP/6-31G (d, p) level of theory.

**NMR Spectral Characterization**


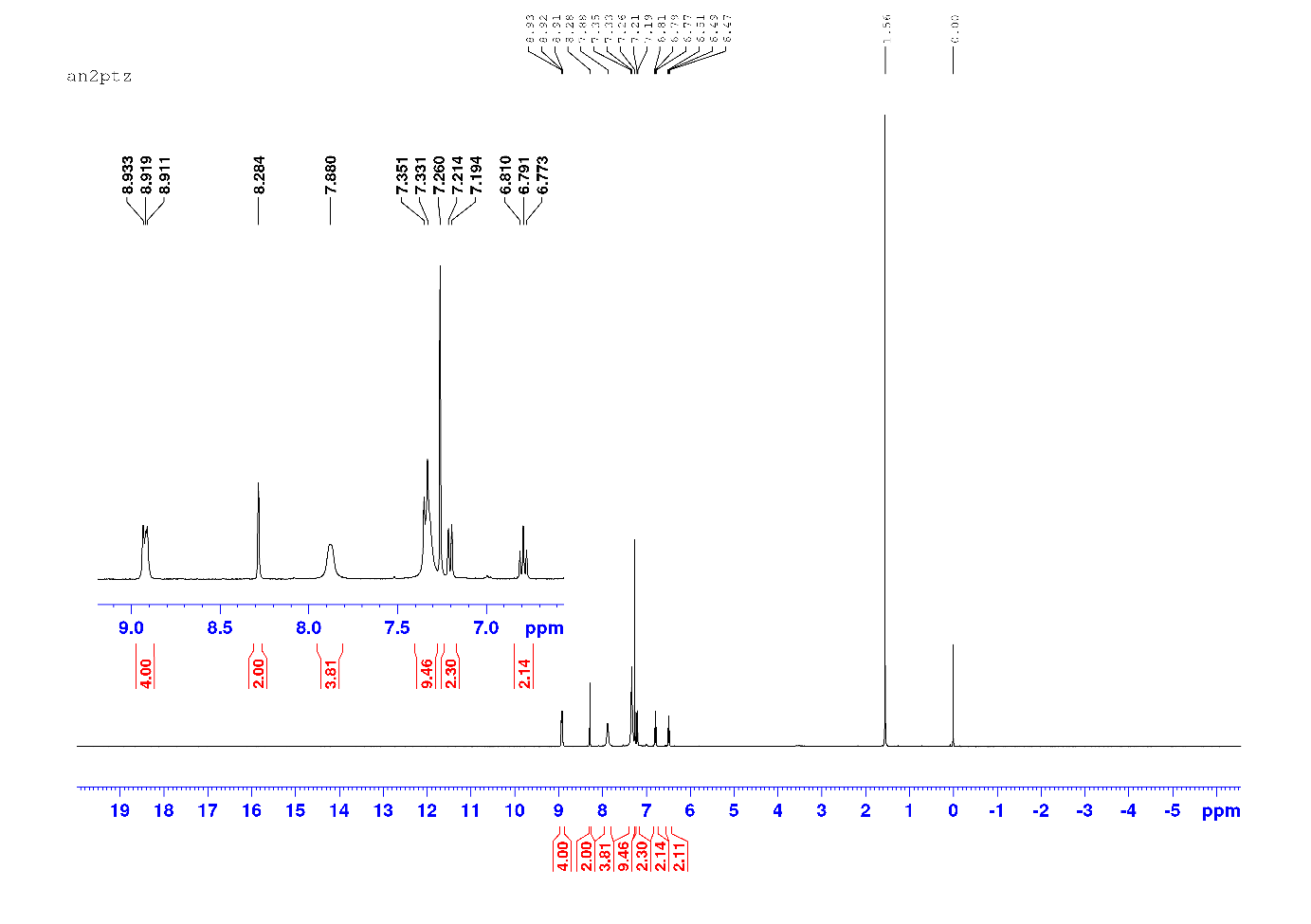


**Figure S1.** ^1^H NMR spectrum of compound **1**

**
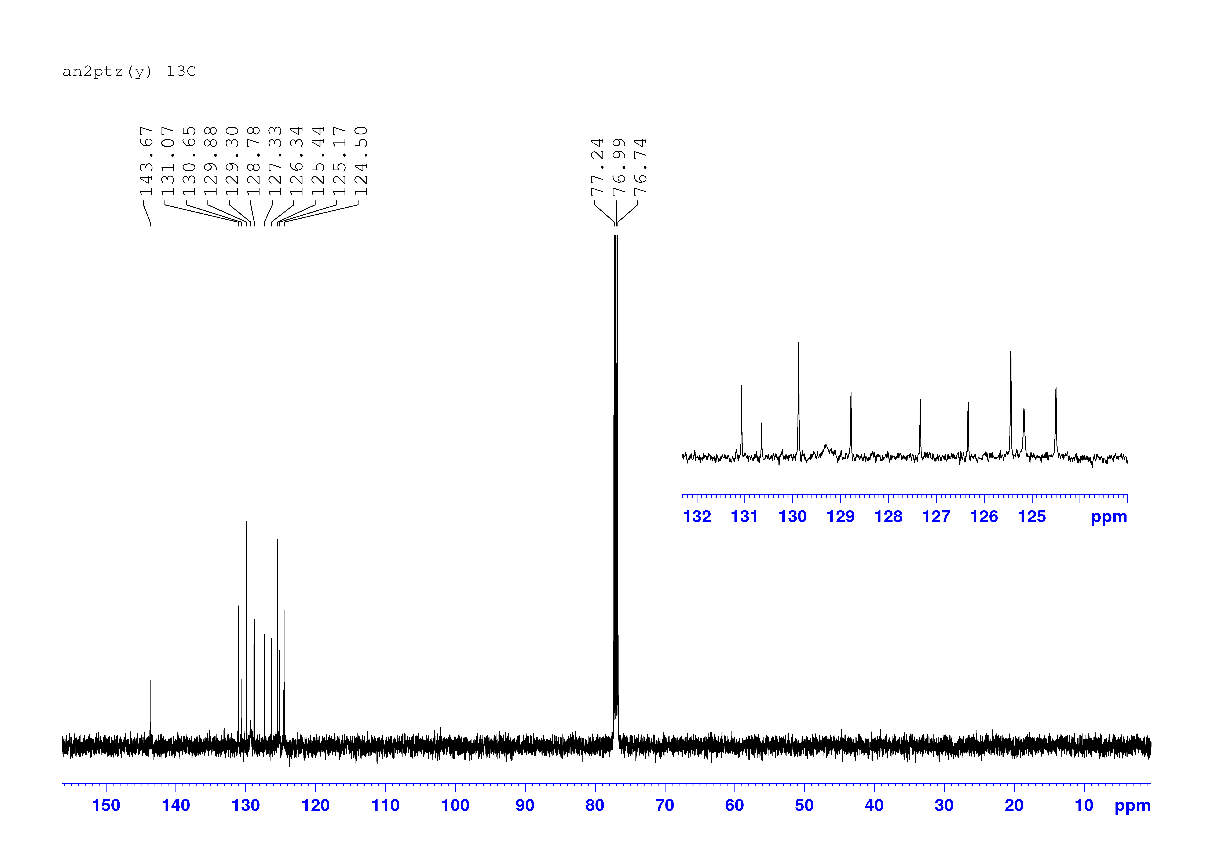
**

**Figure S2. ^13^**C NMR spectrum of compound **1**


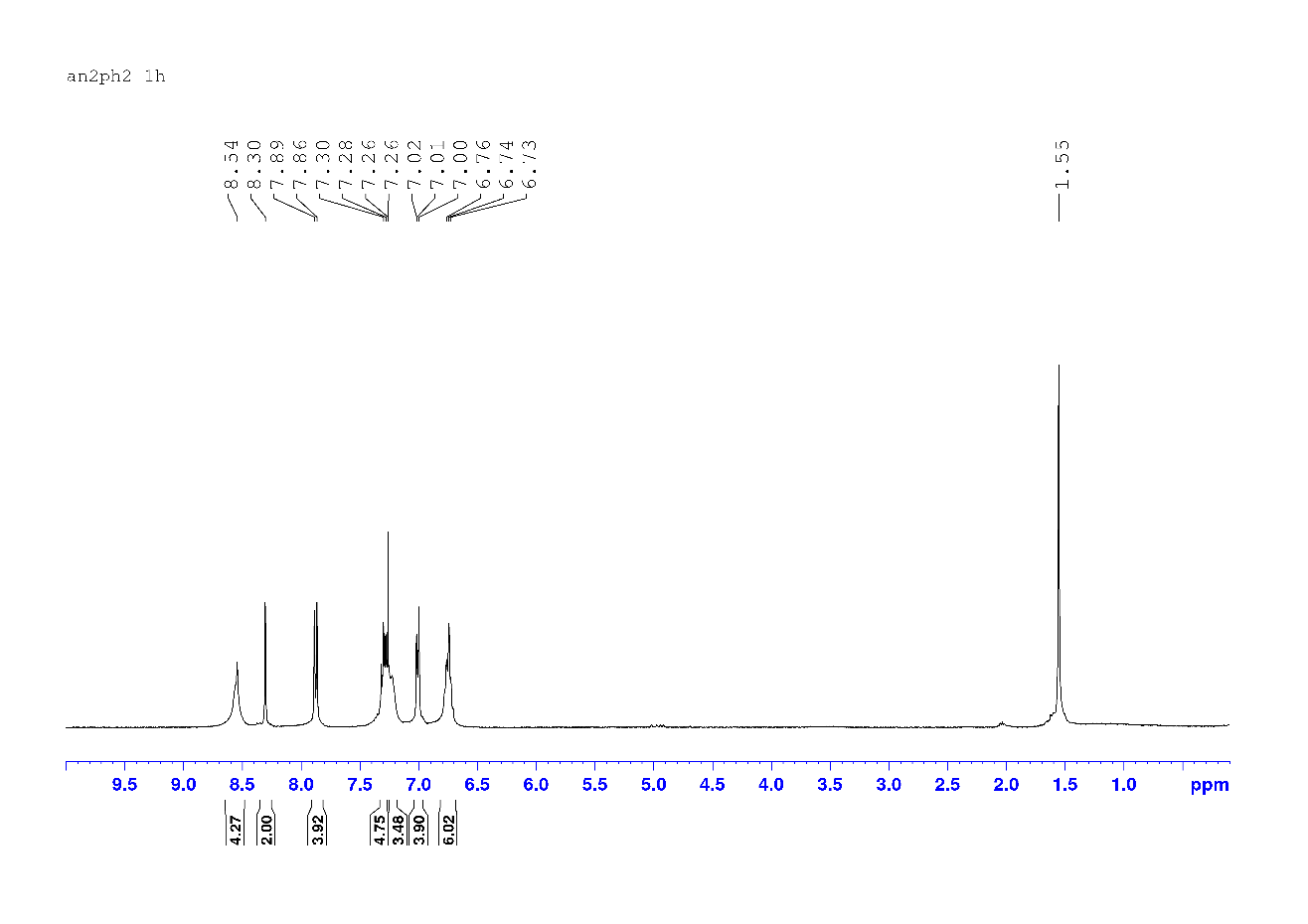


**Figure S3.** ^1^H NMR spectrum of compound **2**


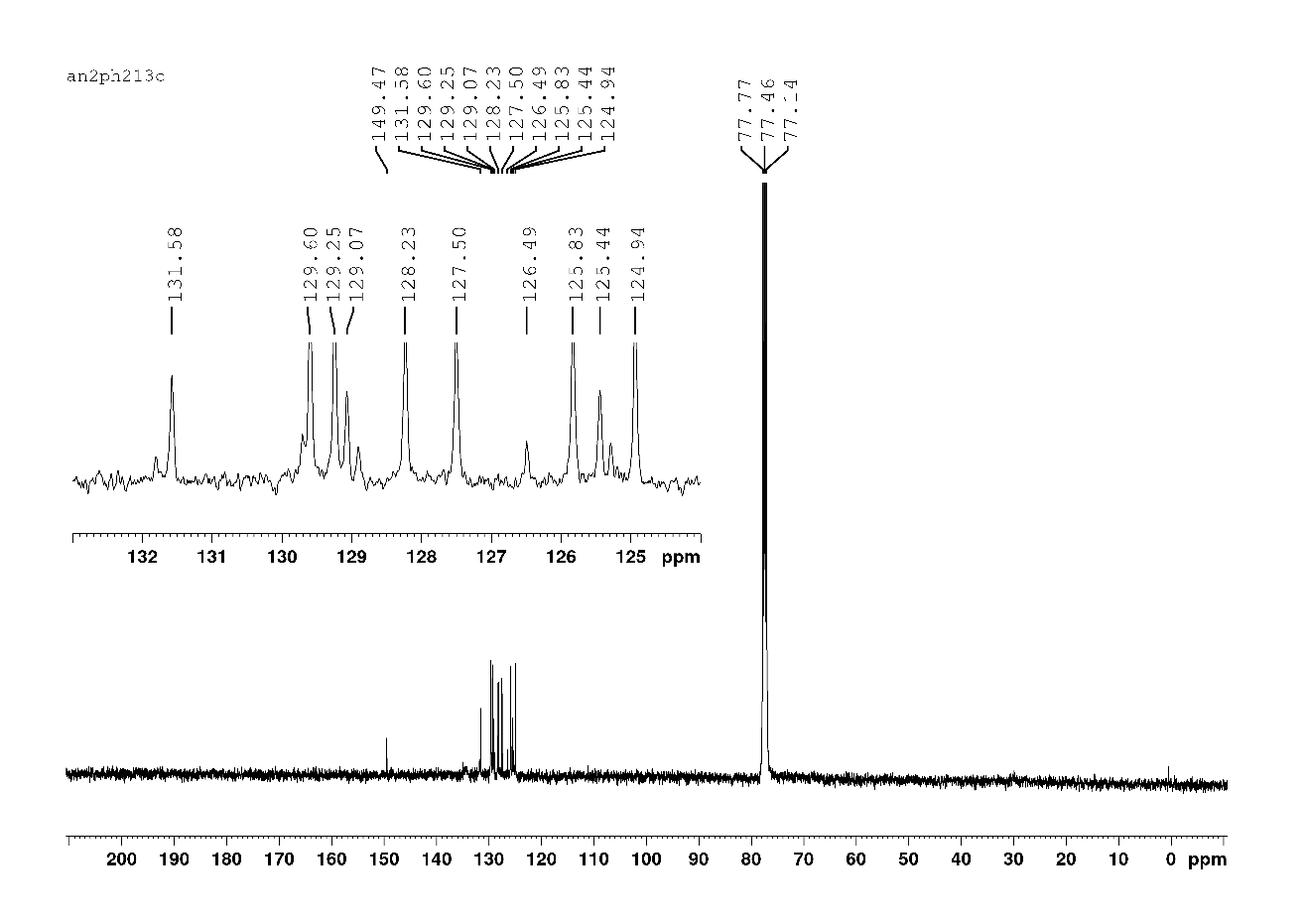


**Figure S4.** ^13^C NMR spectrum of compound **2**

**HRMS Characterization**


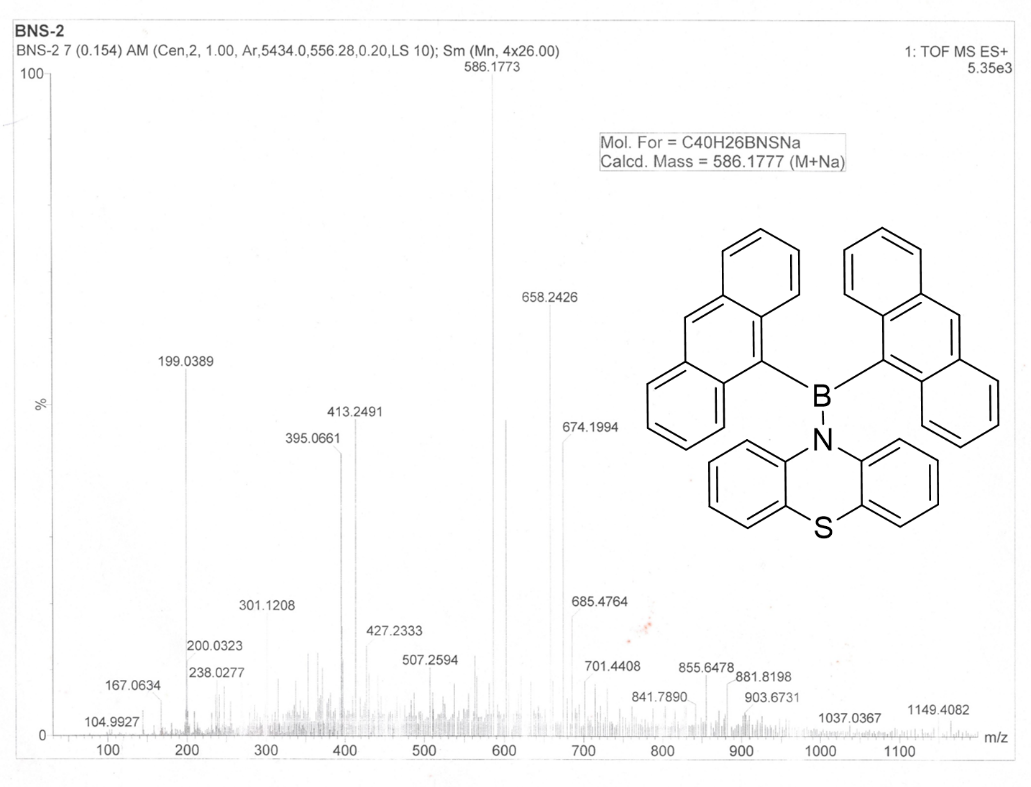


**Figure S5.** HRMS of compound **1**


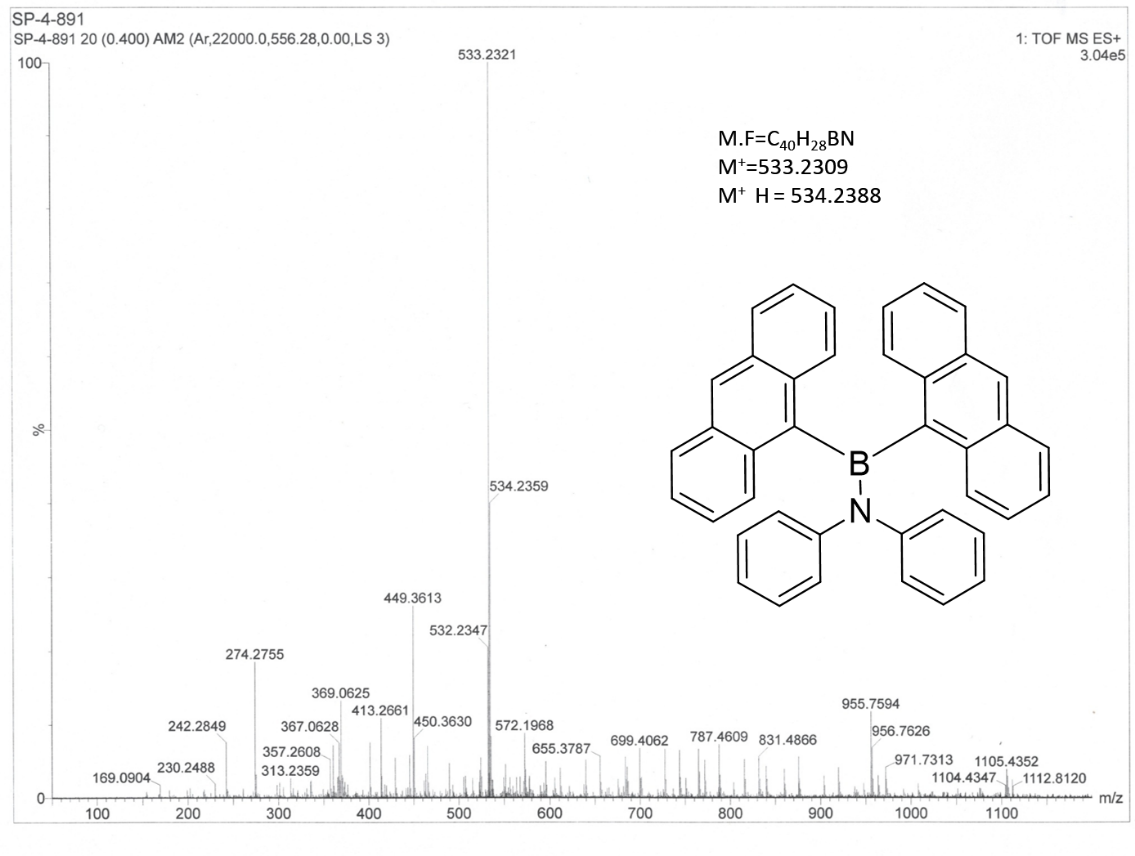


**Figure S6.** HRMS of compound **2**

**Crystallographic Details**

**
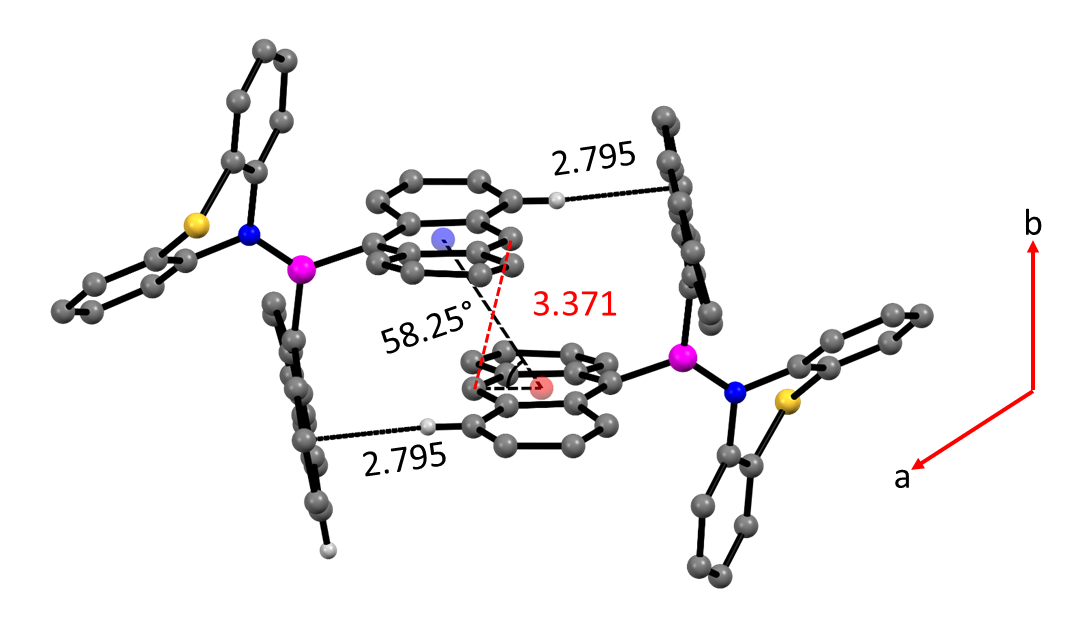
**

**Figure S7.** (A) Crystal structure of **1** depicting intermolecular (C-H....π), π....π Interactions (red dotted lines) and slip angle between two anthryl planes of different molecules. (B) The crystallographic axis along the picture is given.


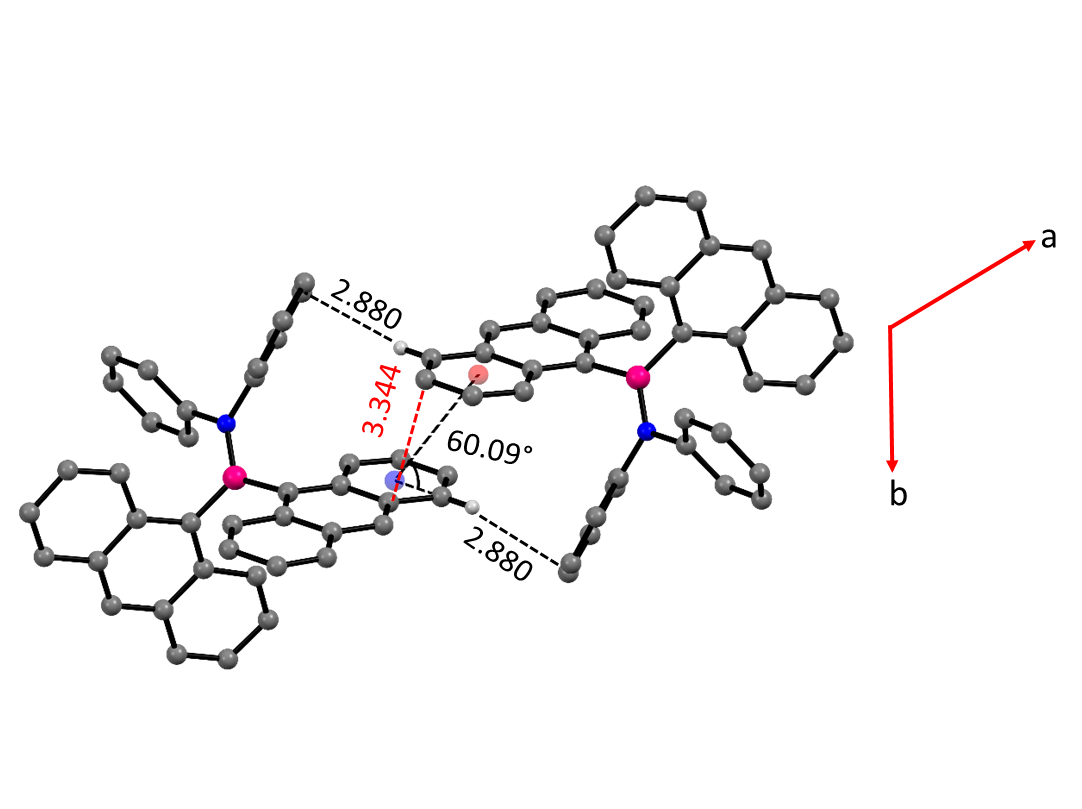


**Figure S8.** Crystal structure of **2** depicting intermolecular (C-H....π) π....π Interactions (red dotted lines) and slip angle between two anthryl planes of different molecules. (B) The crystallographic axis along the picture is given.

**Table S1.** Single crystal structure refinement data of **1** and **2.**

| Identification code | **1** | **2** |
| --- | --- | --- |
| Empirical formula | C_40_ H_26_ B N S | C_40_ H_28_ B N |
| Formula weight | 563.49 | 533.44 |
| Temperature (K) | 100(2) | 100(2) |
| Wavelength (Å) | 0.71073 | 0.71073 |
| Crystal system | Monoclinic | Triclinic |
| Space group | P 21/n | P -1 |
| Unit cell dimensions (Å) | a = 16.8701(7) | a = 11.3417(7) |
|  | b = 9.2601(4) | b = 11.6036(8) |
|  | c = 18.0288(8) | c = 13.8519(9) |
| Unit cell angle | α= 90° | α= 99.345(3)° |
|  | β= 91.1080(10)° | β= 108.237(4)° |
|  | γ = 90° | γ = 114.742(3)° |
| Volume (Å^3^) | 2815.9(2) | 1478.73(17) |
| Z | 4 | 2 |
| Density (calculated Mg/m3) | 1.329 | 1.198 |
| Absorption coefficient mm^-1^ | 0.147 | 0.068 |
| F(000) | 1176 | 560 |
| Theta range for data collection | 3.154 to 27.567°. | 1.646 to 30.613°. |
| Index ranges | -21<=h<=21,  -12<=k<=12,  -23<=l<=23 | -16<=h<=16,  -16<=k<=16,  -19<=l<=19 |
| Reflections collected | 81310 | 33910 |
| Independent reflections | 6470 [R(int) = 0.0584] | 9033 [R(int) = 0.0350] |
| Completeness to theta = | 99.8 % (25.242°) | 100.0 % (25.242°) |
| Refinement method | Full-matrix least-squares on F^2^ | Full-matrix least-squares on F^2^ |
| Data / restraints / parameters | 6470 / 0 / 492 | 9033 / 0 / 379 |
| Goodness-of-fit on F2 | 0.967 | 0.848 |
| Final R indices [I>2sigma(I)] | R1 = 0.0397, wR2 = 0.0945 | R1 = 0.0531, wR2 = 0.1808 |
| R indices (all data) | R1 = 0.0589, wR2 = 0.1040 | R1 = 0.0729, wR2 = 0.2148 |
| Largest diff. peak and hole e.Å^-3^ | 0.392 and -0.299 | 0.521 and -0.282 |
| CCDC Number | 1954827 | 1952521 |

**Optical properties**

**
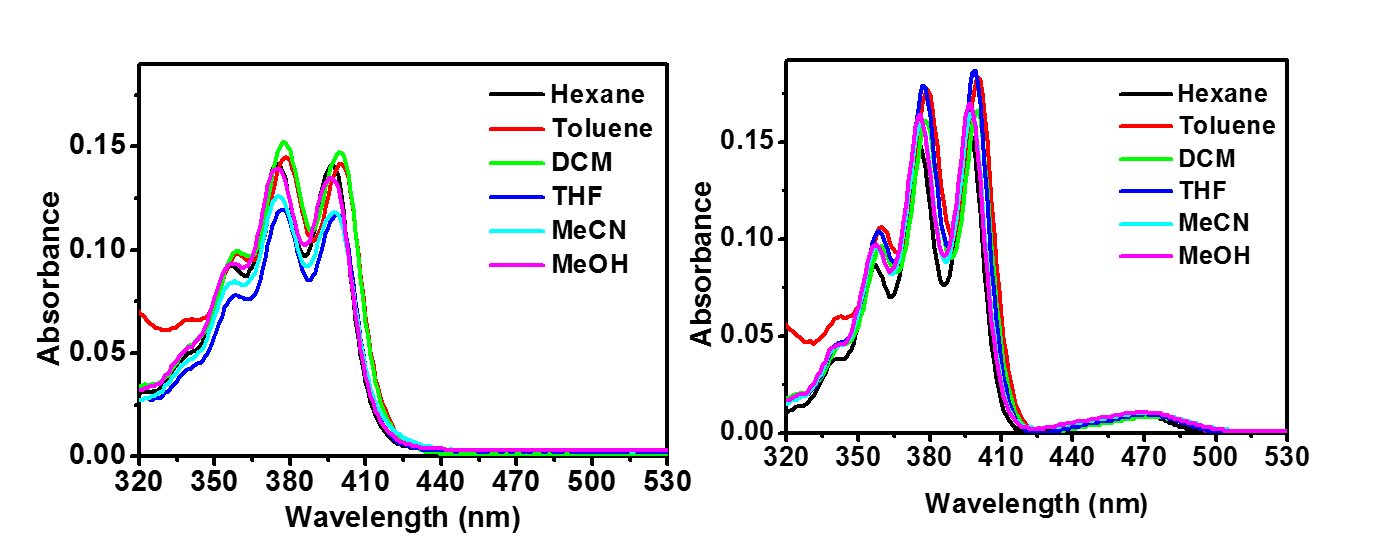
**

**Figure S9.** UV-Vis spectra of **1** (left) and **2** (right) in solvent of different polarity (Conc. 10^-5^ M)

**Table S2.** Absorption maxima of individual vibrational bands (λ_max_) and corresponding ε for **1** and **2.**

| **Solvent** | **λ_max_ (ε)** | **λ_max_ (ε)** | **λ_max_ (ε)** | **λ_max_ (ε)** | **λ_max_ (ε)** | **λ_max_ (ε)** | **λ_max_ (ε)** |
| --- | --- | --- | --- | --- | --- | --- | --- |
|  | **1** | | | **2** | | | |
| **Hexane** | 358  (9200) | 375  (14212) | 397  (14110) | 357  (8735) | 376  (14900) | 397  (15565) | 477  (1070) |
| **Toluene** | 359  (9800) | 378  (14535) | 400  (14184) | 360  (10660) | 379  (17796) | 400  (18444) | 474  (890) |
| **DCM** | 359  (10000) | 378  (15210) | 400  (14720) | 359  (9667) | 378  (16193) | 400  (16659) | 470  (1009) |
| **THF** | 358  (7800) | 376  (11903) | 399  (11708) | 358  (10530) | 377  (17939) | 399  (18772) | 470  (1008) |
| **MeCN** | 358  (8500) | 376  (12620) | 398  (11818) | 358  (9710) | 376  (16112) | 397  (16597) | 470  (1190) |
| **MeOH** | 358  (9300) | 375  (13908) | 396  (13512) | 357  (9766) | 376  (16540) | 397  (17088) | 470  (991) |


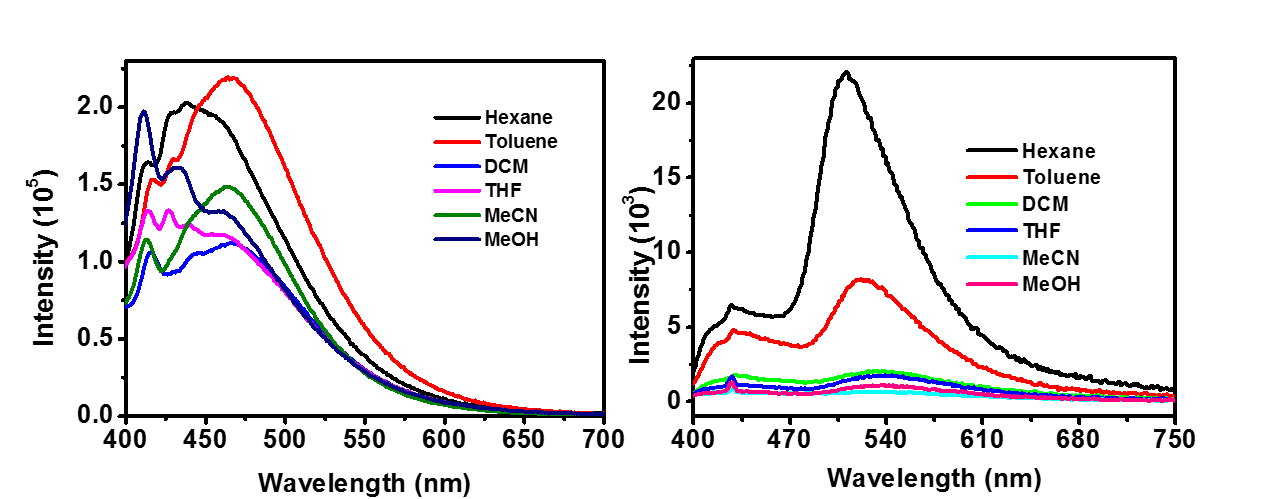


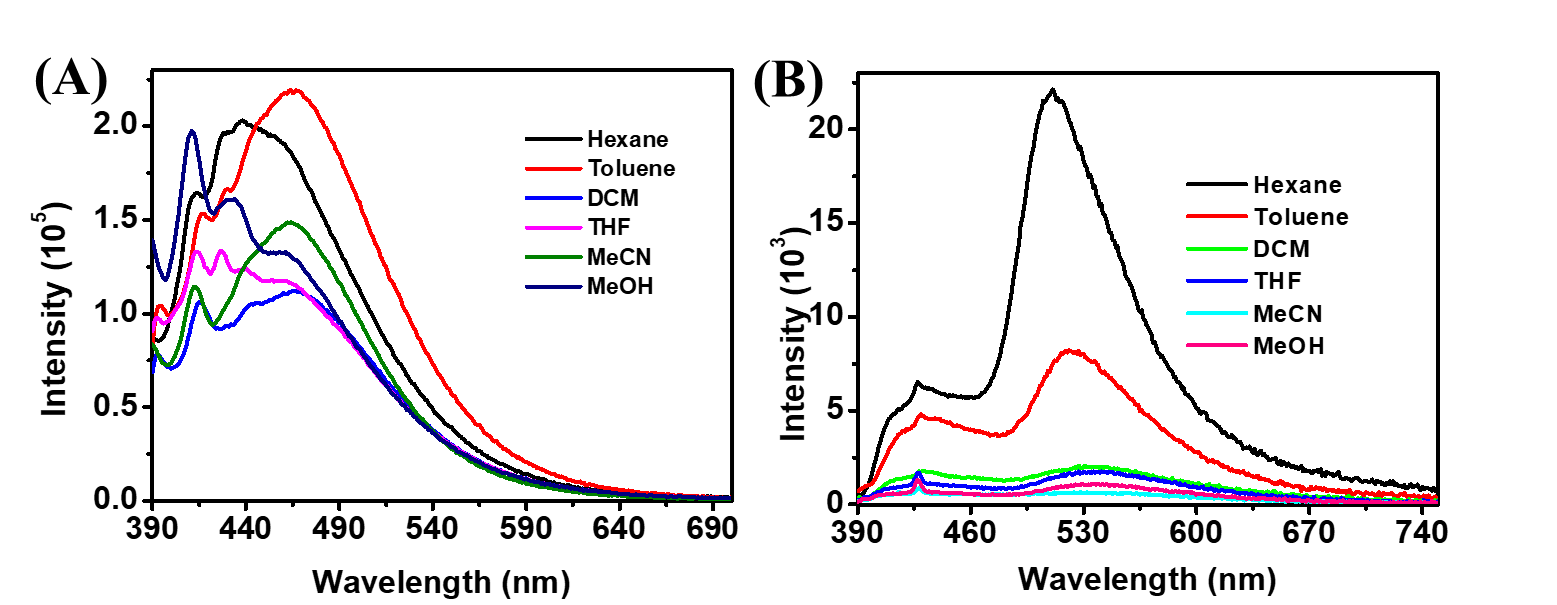


**Figure S10.** Photoluminescence spectra of **1** (left) and **2** (right) in solvent of different polarity (Conc. 10^-5^ M; λ_ex_ = 380 nm).

**Table S3.** time resolved decay kinetics data of **1** (Left) and **2** (Right)

| Solvent | **1** | **2** | |
| --- | --- | --- | --- |
|  | 450 nm | 430nm | 550 nm |
| Hexane | τ _1_ = 0.68 ns (6.82%)  τ _2_ = 8.09 ns (93.18%)  χ^2^ =1.12 | τ _1_= 0.70 ns (82.01%)  τ _2_= 4.7 ns (17.99%)  χ^2^  =1.26 | τ _1_= 0.68 ns (17.90%)  τ _2_= 8.4 ns, (82.10%)  χ^2^  =1.17 |
| THF | τ _1_ = 0.56ns (27.60%)  τ _2_ = 8.31 ns (72.40%)  χ^2^ =1.11 | τ _1_= 0.87 ns (80.61%)  τ _2_= 6.9 ns (19.39%)  χ^2^  =1.21 | τ _1_= 0.60 ns (16.30%)  τ _2_= 4.07 ns (83.70%)  χ^2^  =1.14 |
| MeOH | τ _1_ = 0.45ns (38.47%)  τ_2_= 6.54 ns (61.53%)  χ^2^ =1.24 | τ _1_= 0.98ns (85.17%)  τ _2_= 6.51ns (14.83 %)  χ^2^  =1.15 | τ _1_= 0.77ns (22.55) %  τ _2_= 5.10 ns (11.45%)  χ^2^ =1.13 |

**Table S4.** Excited state lifetime of **2** in THF and aggregates in THF/Water mixture (10:90 %)

|  | Prompt |  | Delayed |  |
| --- | --- | --- | --- | --- |
| THF | τ_1_= 0.87 ns,( 80.61%)  τ_2_= 6.9 ns, (19.39%) | χ^2^=1.01 | - |  |
| Aggregate  (Water:THF 9:1) | 430nm  τ_1_= 4.28 ns (45.06%)  τ_2_= 7.37 ns (54.94%)  520 nm  τ_1_ = 5.9 ns (95.05%)  τ_2_ = 14.66 ns (5.95%) | χ^2^=1.12  χ^2^=1.11 | 520 nm  τ_1_= 4.85μs (60.48%)  τ_2_= 7.7μs (39.52%) | χ^2^=1.21 |

**Table S5.** Temperature dependent DF lifetime of **1** and **2**

|  | Temp | Lifetime at λ_max_ (in Toluene) | χ^2^ | Lifetime at λ _max_  (in solid) | χ^2^ |
| --- | --- | --- | --- | --- | --- |
| **1** | 80K | 435 nm  τ = 5.7 μs 100%) | 1.12 | 530 nm  τ _1_=8.3μs (98.11%),  τ _2_ = 17.14μs (1.89 %), | 0.98 |
|  | 200K | 475 nm  τ _1_ = 3.2 μs (27.10%)  τ _2_ = 7.9 μs (72.9%) | 1.26 | - |  |
|  | 300K | 470 nm  τ _1_ = 5.4 μs (54%)  τ _2_ = 7.06 μs (45%) | 1.18 | 530 nm  τ _1_ = 8.1 μs (98.91%),  τ _2_ = 0.2 μs (1.09%) | 1.21 |
| **2** | 80K | 430 nm  τ _1_= 6.20 μs (98.99%),  τ _2_= 9.10 μs (1.01%), | 1.13 | 530 nm  τ _1_ = 6.35 μs,(96.36%),  τ _2_ = 36.54μs (3.64 %), | 1.24 |
|  | 300K | 530 nm  τ _1_ = 5.90 μs (79.79%)  τ _1_ = 7.79 μs (20.21%)  430 nm  τ _1_= 5.81 μs 95.50%,  τ _2_= 6.12 μs 4.50%. | 1.17 | 530 nm  τ _1_= 6.21 μs 97.38%,  τ _2_= 27.19 μs 2.62%) | 1.22 |

**Table S6.** Temperature dependent TCSPC lifetime of **1** and **2.**

|  | Temp | Lifetime at λ_max_ (in Toluene) | χ^2^ | Lifetime at λ _max_  (in solid) | χ^2^ |
| --- | --- | --- | --- | --- | --- |
| **1** | 80K | 435 nm  (τ _1_= 8.8 ns 100%) | 1.1 | 530 nm    τ _1_= = 4.61 ns (23.30%),  τ _1_= = 1.02 ns (76.70 %), | 1.06 |
|  | 200K | 475 nm  τ _1_= 4.1 ns (5.54 %,)  τ _2_= 12.2 ns (94.36 %) | 1.01 |  |  |
|  | 300K | 470 nm  τ _1_= 1.51 ns (29.59%)  τ _2_= 8.41 ns (70.41%) | 1.21 | 530 nm  τ _1_= 0.8 ns (63.59%),  τ _2_= 5.1 ns (36.42%) | 1.12 |
| **2** | 80K | 430 nm  τ _1_= 2.90 ns (96.95%),  τ _2_= 9.10 ns (3.05%), | 1.12 | 430 nm  τ _1_= 2.28ns, (63.25%),  τ _2_= 5.59 ns (36.75 %)  570 nm  τ _1_= 9.85 ns, (97.23%),  τ _2_= 51.45 ns (2.77 %) | 0.94  1.23 |
|  | 200K | 530 nm  τ _1_= = 3.22 ns 23.38%,  τ _2_= = 7.19 ns 76.62%) | 1.09 |  |  |
|  | 300K | 530 nm  τ _1_= = 1.51 ns (12.59%)  τ _2_= = 7.41 ns (87.41%) | 1.13 | 430 nm  τ _1_= 2.50ns, (52.25%),  τ _2_= 6.69 ns (47.75 %)  530 nm  τ _1_= 6.08 ns, (47.04%),  τ _2_= 10.31 ns (52.96 %) | 1.07  1.04 |

**Figure S11.** Effect of O_2_ on DF of in **1** (left) and **2** (right) (λ_ex_ = 380 nm)

**
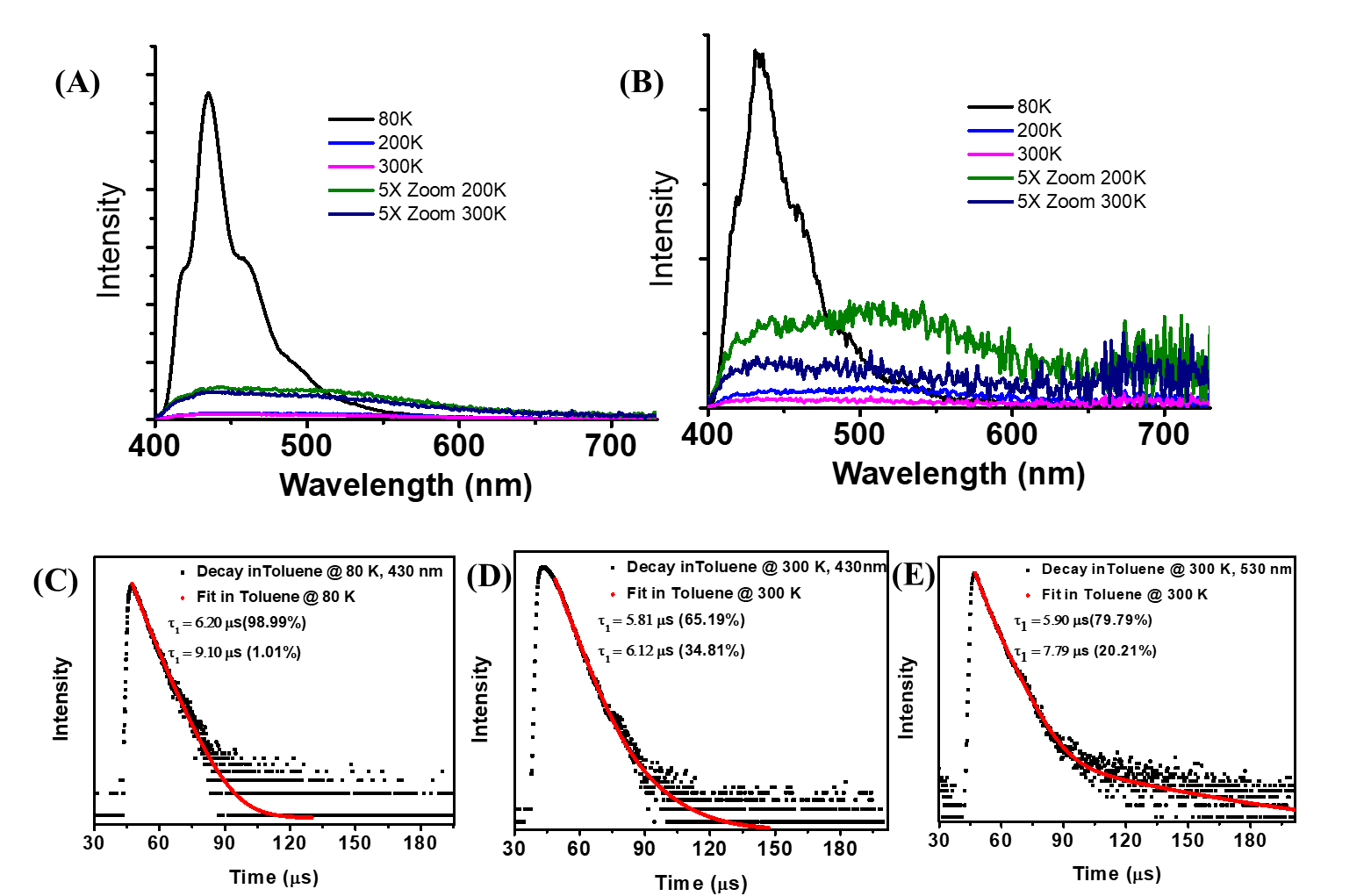
**

**Figure S12.** Prompt (A) and delayed (B) spectra of **2** at 80, 200K, and 300K and 5X zoom image of 200K, and 300K emission. (λ_ex_ = 380 nm; (Delay time 30 μs)) Excited state decay kinetics fitting for the DF of solution of **2** at 80K (Emission = 430 nm) (C) and 300K (Emission =430 nm (D) and 530 nm (E)) in toluene.

**Theoretical studies**

**Table S7**. Frontier molecular orbitals of **1** and calculated possible electronic transitions at B3LYP/631G (H = HOMO; L = LUMO).

|  | **Excitation energies and oscillator strengths** | |
| --- | --- | --- |
| 1 singlet | S1, 2.7688 eV (447.79 nm) f=0.0048 | (H→L) (97.15%) |
|  | S2, 3.0118 eV (411.67 nm) f=0.0540 | (H-1→L) (8.23%)  (H-1→L+1) (43.59%)  (H→L+1) (43.59%) |
|  | S3, 3.0664eV (404.33 nm) f=0.1032 | (H-1→L) (27.366%)  (H-1→L+1) (47.20%)  (H→L+1) (22.18%) |
| 1-Triplet | T1, 1.7176eV (721.85 nm) f=0.0000 | (H-1→L+1) (3.70%)  (H→L+1) (90.72%) |
|  | T2,1.7387 eV, (713.10 nm) f=0.0000 | (H-1→L) (88.30%)  (H→L) (6.74%)  (H-1→L) (3.28%) |
|  | T3, 2.7506 eV 450.75 nm f=0.0000 | (H-1→L) (6.33%)  (H→L) (91.05%) |

**Table S8.** Frontier molecular orbitals of **2** and calculated possible electronic transitions at B3LYP/631G (H = HOMO; L = LUMO).

|  | Excitation energies and oscillator strengths: | |
| --- | --- | --- |
| 2 singlets | S1, 2.5835 eV (479.91 nm) f=0.0822 | (H→L) (93.13%)  (H-1→L+1) (6.34%) |
|  | S2, 2.6472 eV (468.36 nm) f=0.0159 | (H-1→L) (76.39%)  (H→L+1) (23.11%) |
|  | S3, 2.9415 eV (421.50 nm) f=0.1012 | (H-1→L+1) (92.86%)  (H→L) (6.22%) |
| 2 Triplets | T1, 1.7189 eV (721.28 nm) f=0.0000 | (H→L) (45.09%)  (H-1→L+1) (51.467%) |
|  | T2 1.7245 eV (718.97 nm) f=0.0000 | (H-1→L) (51.95%)  (H→L+1) (44.44%) |
|  | T3, 2.8647 eV 432.80 nm f=0.0000 | (H-1→L+1) (49.94%)  (H→L) (46.85%) |

**
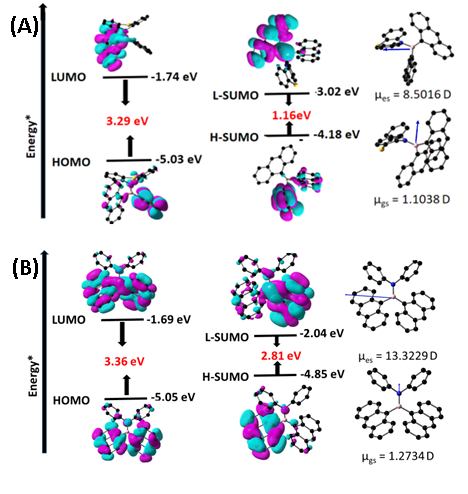
**

**Figure S13.** Frontier molecular orbitals of **1** and **2 (**A) and (B) respectively in ground state (left) and first excited state (right).


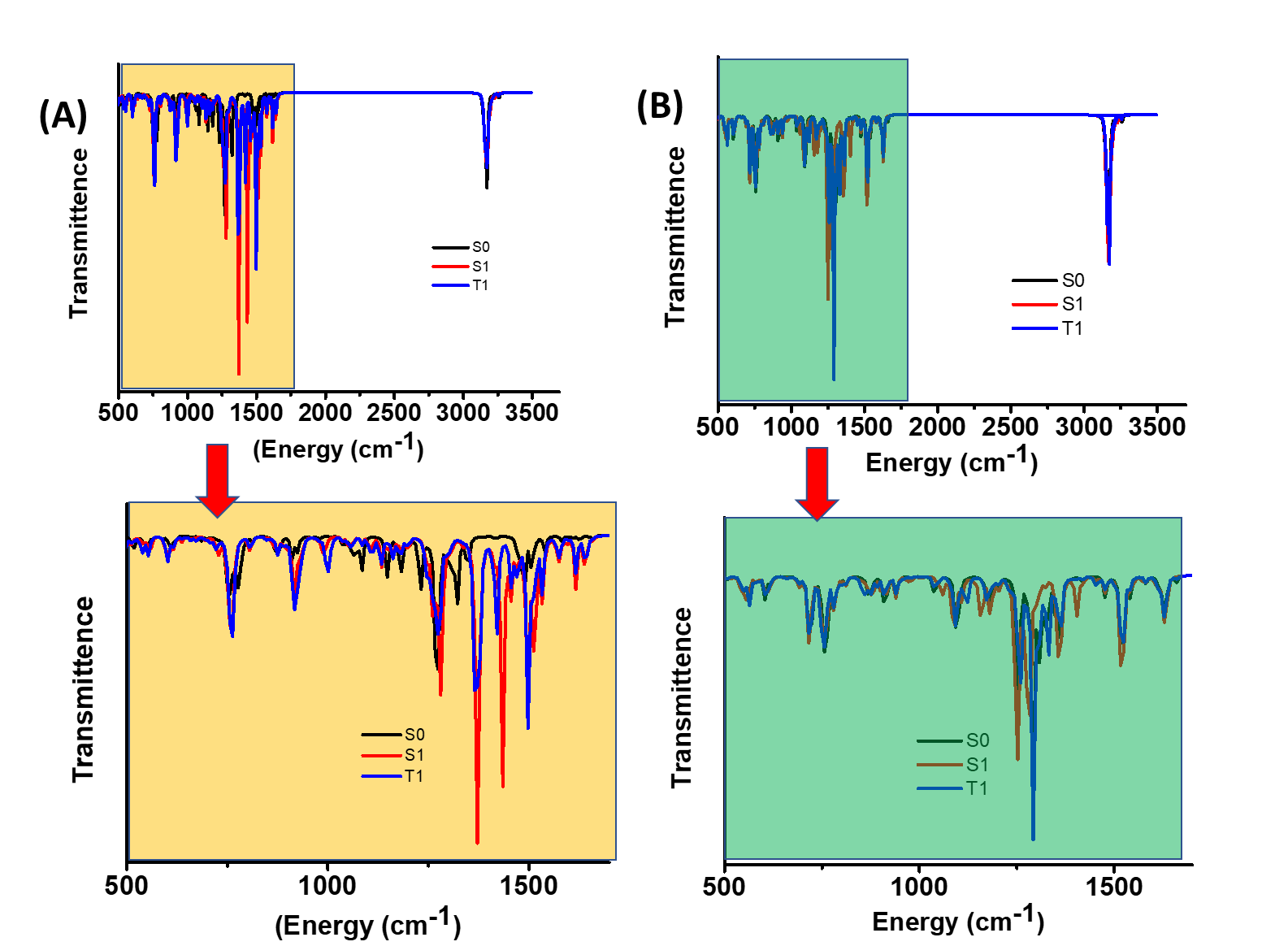


**Figure S14.** DFT Calculated IR spectra of **1** (A) and **2** (B) in S_0_, S_1_ and T_1_ states.

**Delayed emission measurements**

**S15.** The prompt and delayed fluorescence of solids of **1** at 300 K. (λ_ex_ = 380 nm; Delay time 30 μs)


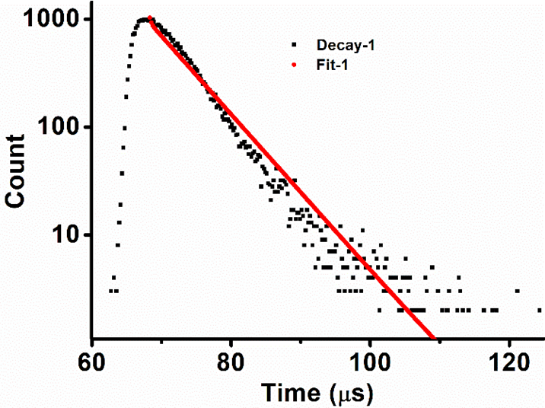

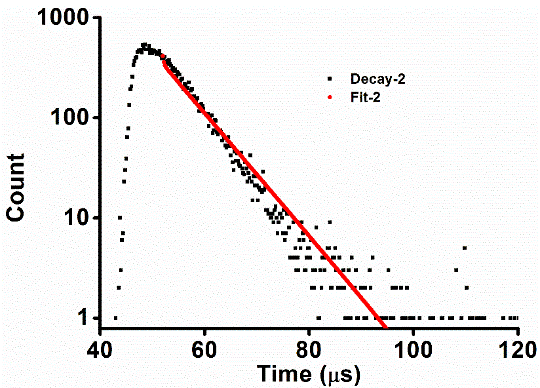


**Figure S16.** Excited state decay kinetics fitting for the DF of solids of **1** (left) and **2** (right) at 300K.


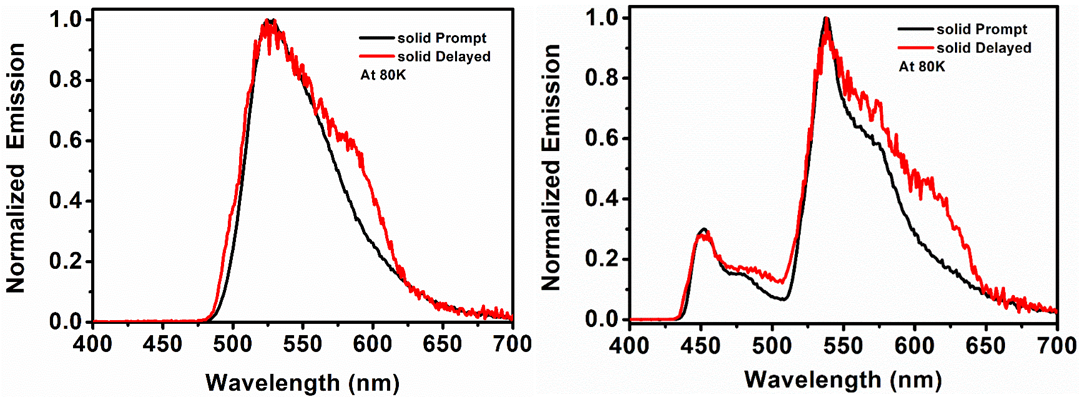


**Figure S17.** The prompt and delayed fluorescence of solids of **1** (left) and **2** (right) (λ_ex_ = 380 nm; Delay time 30 μs)

.

**
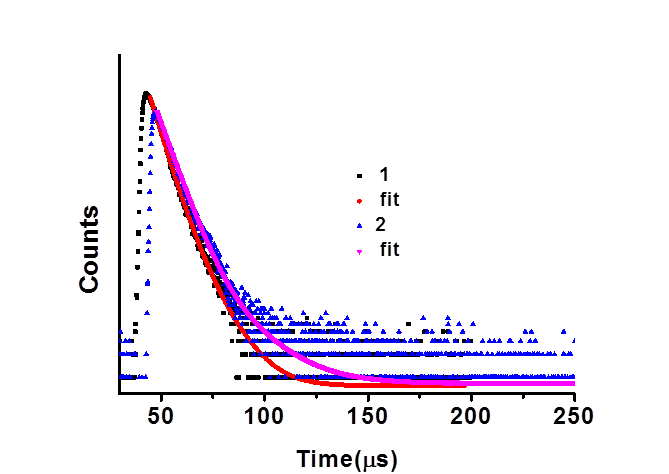
**

**Figure S18.** Excited state decay kinetics fitting for the DF of solids of **1** and **2** at 80 K.


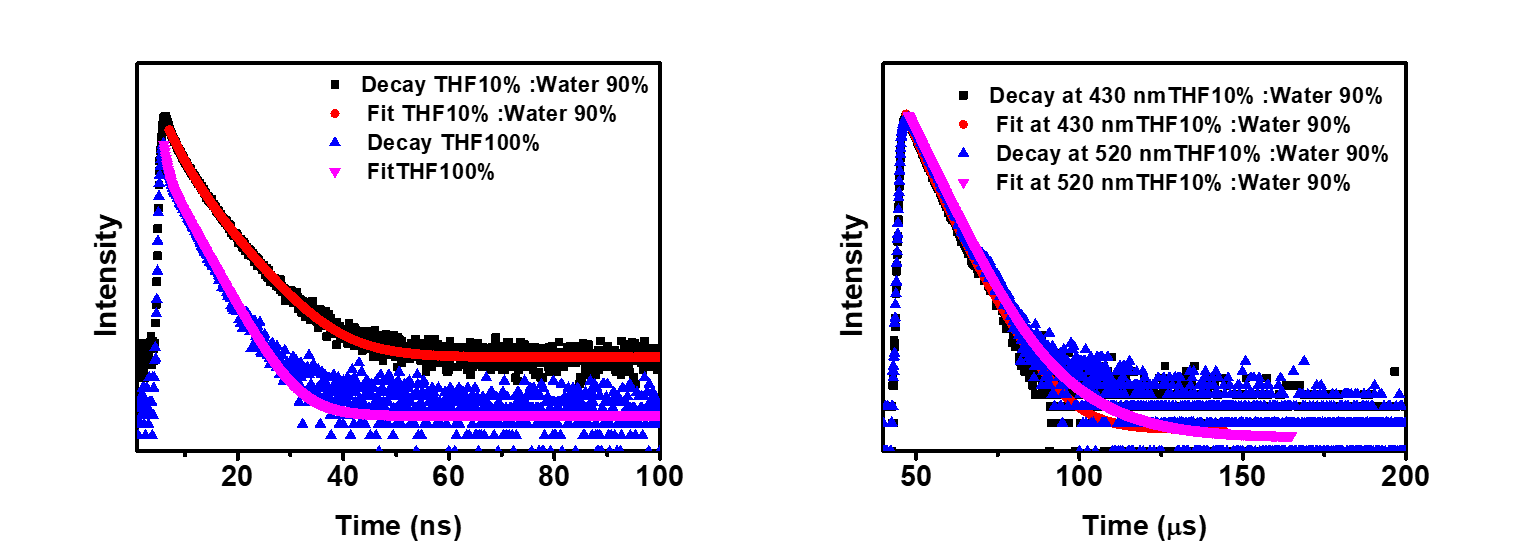


**Figure S19.** Excited state decay kinetics fitting for the prompt (left) and DF (right) of aggregates of **2**.

**Cartesian coordinates of optimized structures**

**Table S9.** Cartesian coordinates of optimized structures in S_0_ for **1,** calculated using Gaussian 09 at the B3LYP/6-31G (d,p) level of theory.

Center Number Atomic Number Atomic Type Coordinates (Angstroms)

X Y Z

---------------------------------------------------------------------

1 6 0 2.977310 -1.863129 3.400545

2 6 0 3.715238 -0.804912 2.979492

3 6 0 3.242194 0.007422 1.943728

4 6 0 2.008953 -0.282384 1.351836

5 6 0 1.272836 -1.384787 1.788395

6 6 0 1.744717 -2.159638 2.793981

7 6 0 3.993963 1.106105 1.504101

8 6 0 1.511559 0.533042 0.333414

9 6 0 2.250439 1.641384 -0.094238

10 6 0 3.491058 1.927506 0.483112

11 6 0 4.219794 3.035899 0.031613

12 1 0 5.172729 3.262710 0.461299

13 6 0 3.715449 3.822684 -0.954043

14 6 0 2.466168 3.533820 -1.527880

15 6 0 1.747415 2.465484 -1.106258

16 1 0 4.946862 1.316041 1.947189

17 1 0 3.337273 -2.474443 4.200718

18 1 0 4.657086 -0.590347 3.442112

19 1 0 0.335812 -1.614783 1.331402

20 1 0 1.174818 -3.001259 3.128146

21 1 0 4.272563 4.670079 -1.297405

22 1 0 2.080612 4.160811 -2.303814

23 1 0 0.795369 2.251940 -1.546311

24 6 0 -1.317524 -1.332700 -1.751127

25 6 0 1.073932 -1.588893 -1.795780

26 6 0 -1.903254 -1.776371 -0.557813

27 6 0 -1.962230 -1.562734 -2.973412

28 6 0 1.591420 -2.165768 -0.630040

29 6 0 1.607262 -1.929867 -3.044197

30 6 0 -3.133945 -2.449135 -0.586138

31 6 0 -3.193549 -2.232575 -3.003019

32 1 0 -1.514391 -1.224349 -3.884018

33 6 0 2.635794 -3.094598 -0.715188

34 6 0 2.653391 -2.856223 -3.130399

35 1 0 1.214848 -1.482364 -3.933811

36 6 0 -3.776991 -2.680621 -1.809395

37 1 0 -3.580153 -2.783798 0.325637

38 1 0 -3.687700 -2.400479 -3.936340

39 1 0 3.029457 -3.537798 0.174631

40 6 0 3.166346 -3.442667 -1.965751

41 1 0 3.060641 -3.115820 -4.087141

42 1 0 -4.713445 -3.199415 -1.833621

43 1 0 3.962315 -4.156205 -2.031592

44 6 0 -1.344878 0.751867 0.294034

45 6 0 -2.080112 -0.051685 1.180507

46 6 0 -1.808534 2.022442 -0.045751

47 6 0 -3.294472 0.414804 1.697220

48 6 0 -1.615072 -1.320800 1.547319

49 6 0 -3.019869 2.490790 0.467604

50 6 0 -1.058631 2.828324 -0.905958

51 6 0 -4.036697 -0.400709 2.560585

52 6 0 -3.764097 1.691039 1.346489

53 6 0 -2.353029 -2.099791 2.377967

54 1 0 -0.678219 -1.676966 1.172953

55 6 0 -3.478711 3.760678 0.094245

56 6 0 -1.517006 4.054743 -1.254588

57 1 0 -0.121243 2.475826 -1.287348

58 6 0 -3.578706 -1.635933 2.885983

59 1 0 -4.965369 -0.052315 2.961657

60 1 0 -4.686769 2.052191 1.746088

61 1 0 -1.999592 -3.073889 2.645917

62 1 0 -4.406863 4.129822 0.474312

63 6 0 -2.742185 4.525280 -0.753937

64 1 0 -0.940507 4.665923 -1.916284

65 1 0 -4.154538 -2.260293 3.533441

66 1 0 -3.096876 5.492371 -1.042663

67 1 0 1.188212 -1.899719 0.323833

68 1 0 -1.410551 -1.601811 0.376455

69 5 0 0.058165 0.206613 -0.374215

70 7 0 -0.028240 -0.621295 -1.716847

**Table S10.** Cartesian coordinates of optimized structures in S_1_ for **1** calculated using Gaussian 09 at the B3LYP/6-31G (d,p) level of theory.

---------------------------------------------------------------------

Center Number Atomic Number Atomic Type Coordinates (Angstroms)

X Y Z

---------------------------------------------------------------------

1 6 0 -1.735781 -3.711388 3.156444

2 6 0 -2.793373 -3.441265 2.295386

3 6 0 -2.703381 -2.434807 1.298125

4 6 0 -1.486541 -1.677168 1.164945

5 6 0 -0.438399 -1.973241 2.074903

6 6 0 -0.552893 -2.971103 3.045282

7 6 0 -3.783855 -2.159281 0.436296

8 6 0 -1.366687 -0.651830 0.162932

9 6 0 -2.498608 -0.396996 -0.705154

10 6 0 -3.710572 -1.163437 -0.560990

11 6 0 -4.806454 -0.919069 -1.430339

12 1 0 -5.707702 -1.510449 -1.290127

13 6 0 -4.740371 0.036925 -2.434439

14 6 0 -3.561060 0.773809 -2.599901

15 6 0 -2.472373 0.557109 -1.756253

16 1 0 -4.700955 -2.734255 0.540139

17 1 0 -1.821088 -4.483641 3.915030

18 1 0 -3.721152 -4.002882 2.368371

19 1 0 0.484146 -1.410791 2.011644

20 1 0 0.273652 -3.172203 3.720094

21 1 0 -5.585509 0.210944 -3.093572

22 1 0 -3.498139 1.516015 -3.390130

23 1 0 -1.570974 1.138843 -1.903004

24 6 0 1.090127 2.356724 -0.523533

25 6 0 -1.091708 2.355535 0.522952

26 6 0 1.628168 3.415482 0.222048

27 6 0 1.618503 2.075955 -1.790061

28 6 0 -1.620060 2.073488 1.789241

29 6 0 -1.630655 3.414364 -0.221933

30 6 0 2.681371 4.171534 -0.289639

31 6 0 2.679532 2.829845 -2.292504

32 1 0 1.198666 1.267166 -2.378096

33 6 0 -2.681978 2.825959 2.292009

34 6 0 -2.684732 4.168972 0.290059

35 1 0 -1.217178 3.644571 -1.198205

36 6 0 3.215050 3.881056 -1.546861

37 1 0 3.090422 4.986147 0.300989

38 1 0 3.078668 2.599080 -3.275876

39 1 0 -3.080983 2.594231 3.275213

40 6 0 -3.218414 3.877116 1.546996

41 1 0 -3.094429 4.983628 -0.300066

42 1 0 4.036624 4.470502 -1.942486

43 1 0 -4.040592 4.465530 1.942900

44 6 0 1.367207 -0.650873 -0.163967

45 6 0 2.498162 -0.395038 0.705196

46 6 0 1.488421 -1.676275 -1.165912

47 6 0 3.710738 -1.160828 0.562126

48 6 0 2.470536 0.558971 1.756493

49 6 0 2.705910 -2.433167 -1.297872

50 6 0 0.441555 -1.973130 -2.077161

51 6 0 4.805658 -0.915658 1.432569

52 6 0 3.785421 -2.156755 -0.435034

53 6 0 3.558312 0.776380 2.601167

54 1 0 1.568697 1.140252 1.902566

55 6 0 2.797351 -3.439713 -2.295010

56 6 0 0.557444 -2.971053 -3.047305

57 1 0 -0.481270 -1.411004 -2.015247

58 6 0 4.738211 0.040263 2.436660

59 1 0 5.707380 -1.506516 1.293131

60 1 0 4.702973 -2.731168 -0.537990

61 1 0 3.494232 1.518540 3.391384

62 1 0 3.725574 -4.000743 -2.367001

63 6 0 1.740852 -3.710676 -3.157131

64 1 0 -0.268260 -3.172611 -3.723060

65 1 0 5.582702 0.214778 3.096522

66 1 0 1.827428 -4.482944 -3.915585

67 1 0 -1.199318 1.264932 2.376942

68 1 0 1.214719 3.644540 1.198596

69 5 0 0.000286 0.133472 -0.000865

70 7 0 -0.000510 1.578819 -0.000533

---------------------------------------------------------------------

**Table S11.** Cartesian coordinates of optimized structures in T_1_ for **1** calculated using Gaussian 09 at the B3LYP/6-31G (d,p) level of theory

---------------------------------------------------------------------

Center Number Atomic Number Atomic Type Coordinates (Angstroms)

X Y Z

---------------------------------------------------------------------

1 6 0 -2.073377 -3.306793 3.453867

2 6 0 -3.039729 -3.148222 2.498405

3 6 0 -2.840840 -2.276221 1.382668

4 6 0 -1.595321 -1.551520 1.252302

5 6 0 -0.620609 -1.750486 2.284597

6 6 0 -0.847008 -2.594215 3.341221

7 6 0 -3.834740 -2.108258 0.416331

8 6 0 -1.383074 -0.663071 0.163611

9 6 0 -2.419757 -0.513625 -0.801067

10 6 0 -3.657125 -1.252122 -0.672453

11 6 0 -4.678985 -1.102544 -1.661900

12 1 0 -5.599218 -1.667710 -1.537475

13 6 0 -4.507270 -0.278318 -2.740110

14 6 0 -3.291500 0.446032 -2.885073

15 6 0 -2.292871 0.332358 -1.952846

16 1 0 -4.768450 -2.658020 0.512774

17 1 0 -2.236547 -3.971036 4.297441

18 1 0 -3.983894 -3.682107 2.570336

19 1 0 0.325311 -1.226524 2.229017

20 1 0 -0.081316 -2.722271 4.100870

21 1 0 -5.289884 -0.175835 -3.485953

22 1 0 -3.156076 1.097758 -3.743415

23 1 0 -1.379529 0.899710 -2.087012

24 6 0 1.115994 2.321309 -0.514858

25 6 0 -1.120319 2.343686 0.421334

26 6 0 1.600057 3.411730 0.221241

27 6 0 1.720789 1.998385 -1.735246

28 6 0 -1.697060 2.104013 1.673784

29 6 0 -1.626272 3.376648 -0.379410

30 6 0 2.680641 4.153246 -0.250163

31 6 0 2.809937 2.738652 -2.197264

32 1 0 1.339543 1.169403 -2.321181

33 6 0 -2.779154 2.870812 2.106897

34 6 0 -2.700219 4.146071 0.062698

35 1 0 -1.173138 3.574389 -1.345177

36 6 0 3.293931 3.818365 -1.459572

37 1 0 3.048379 4.991950 0.333703

38 1 0 3.270919 2.473214 -3.144031

39 1 0 -3.218778 2.670645 3.079524

40 6 0 -3.284636 3.894491 1.305679

41 1 0 -3.085024 4.940349 -0.570226

42 1 0 4.136942 4.397113 -1.824489

43 1 0 -4.122667 4.494158 1.647689

44 6 0 1.374216 -0.663028 -0.147618

45 6 0 2.455068 -0.458779 0.844136

46 6 0 1.581642 -1.647216 -1.227084

47 6 0 3.668551 -1.220600 0.755535

48 6 0 2.333393 0.433560 1.908749

49 6 0 2.799778 -2.403712 -1.294562

50 6 0 0.636605 -1.856046 -2.229857

51 6 0 4.678396 -1.030063 1.698368

52 6 0 3.819331 -2.183307 -0.303715

53 6 0 3.370639 0.619125 2.870513

54 1 0 1.426731 1.017239 2.016361

55 6 0 2.984775 -3.330150 -2.320029

56 6 0 0.836223 -2.805564 -3.277006

57 1 0 -0.293041 -1.301441 -2.222014

58 6 0 4.534517 -0.101220 2.764943

59 1 0 5.593161 -1.610795 1.611658

60 1 0 4.738982 -2.759479 -0.360359

61 1 0 3.225267 1.331477 3.676948

62 1 0 3.909147 -3.901319 -2.351216

63 6 0 1.995005 -3.539969 -3.320025

64 1 0 0.062633 -2.935638 -4.027622

65 1 0 5.339002 0.024661 3.483358

66 1 0 2.167220 -4.270732 -4.104582

67 1 0 -1.299953 1.316859 2.305120

68 1 0 1.125962 3.674038 1.161159

69 5 0 -0.000467 0.117995 -0.006463

70 7 0 -0.002439 1.554194 -0.030146

---------------------------------------------------------------------

**Table S12.** Cartesian coordinates of optimized structures in S_0_ for **2** calculated using Gaussian 09 at the B3LYP/6-31G (d, p) level of theory

---------------------------------------------------------------------

Center Number Atomic Number Atomic Type Coordinates (Angstroms)

X Y Z

---------------------------------------------------------------------

1 5 0 -1.781525 0.032268 0.002238

2 6 0 -2.581559 1.176034 0.756216

3 6 0 -3.590270 0.854698 1.716770

4 6 0 -2.331075 2.558144 0.482729

5 6 0 -3.881921 -0.489674 2.117608

6 6 0 -4.345534 1.897986 2.376155

7 6 0 -3.102195 3.591635 1.141112

8 6 0 -1.364804 3.006942 -0.476584

9 6 0 -4.845312 -0.779343 3.049323

10 1 0 -3.325086 -1.304362 1.679800

11 6 0 -5.344248 1.559503 3.341833

12 6 0 -4.085156 3.233024 2.064646

13 6 0 -2.863089 4.969115 0.839221

14 1 0 -0.764891 2.275642 -0.999803

15 6 0 -1.163897 4.336780 -0.744867

16 6 0 -5.595296 0.256082 3.671040

17 1 0 -5.034495 -1.813909 3.320205

18 1 0 -5.896205 2.368567 3.813000

19 1 0 -4.660379 4.014914 2.555574

20 6 0 -1.919267 5.339989 -0.078574

21 1 0 -3.458576 5.716319 1.357172

22 1 0 -0.416487 4.627282 -1.477507

23 1 0 -6.354783 0.008340 4.406482

24 1 0 -1.746265 6.388122 -0.302749

25 6 0 -2.510474 -1.156401 -0.762093

26 6 0 -3.413933 -0.905648 -1.835218

27 6 0 -2.256697 -2.512801 -0.398012

28 6 0 -3.690121 0.412664 -2.323122

29 6 0 -4.071358 -1.999512 -2.518352

30 6 0 -2.923794 -3.598780 -1.081917

31 6 0 -1.384098 -2.874161 0.680256

32 6 0 -4.555176 0.637066 -3.362248

33 1 0 -3.198747 1.256165 -1.861404

34 6 0 -4.969825 -1.727195 -3.597235

35 6 0 -3.812695 -3.311926 -2.119792

36 6 0 -2.673595 -4.949634 -0.685147

37 1 0 -0.871810 -2.091675 1.225674

38 6 0 -1.170868 -4.180983 1.037226

39 6 0 -5.211718 -0.445952 -4.009691

40 1 0 -4.738850 1.652969 -3.699368

41 1 0 -5.452887 -2.568650 -4.087094

42 1 0 -4.312069 -4.131137 -2.632225

43 6 0 -1.820764 -5.239712 0.344853

44 1 0 -3.185328 -5.744054 -1.222119

45 1 0 -0.498884 -4.411366 1.858801

46 1 0 -5.894984 -0.251044 -4.830846

47 1 0 -1.639792 -6.269196 0.638678

48 6 0 -0.208387 0.048795 0.014742

49 6 0 0.533190 0.656509 1.051046

50 6 0 0.524627 -0.563937 -1.026019

51 6 0 1.924818 0.637839 1.061431

52 1 0 0.005356 1.149758 1.861570

53 6 0 1.917301 -0.561960 -1.041494

54 1 0 -0.014178 -1.045678 -1.836383

55 6 0 2.621755 0.030483 0.010975

56 1 0 2.484020 1.095543 1.871417

57 1 0 2.469536 -1.026620 -1.852299

58 6 0 4.703783 -1.080821 0.638357

59 6 0 4.725399 1.103803 -0.604006

60 6 0 3.936616 -2.105233 1.223788

61 6 0 6.106989 -1.202014 0.706548

62 6 0 6.130566 1.200986 -0.667237

63 6 0 3.977386 2.142802 -1.187674

64 6 0 4.531225 -3.198611 1.851354

65 1 0 2.857241 -2.047334 1.187027

66 6 0 6.691783 -2.301705 1.338352

67 6 0 6.735776 2.293070 -1.292931

68 1 0 2.896946 2.101581 -1.153874

69 6 0 4.592082 3.228172 -1.809751

70 6 0 5.916459 -3.305576 1.914816

71 1 0 3.897707 -3.963902 2.289119

72 1 0 7.775970 -2.365272 1.374975

73 6 0 5.979211 3.311878 -1.868337

74 1 0 7.820999 2.338554 -1.325733

75 1 0 3.973024 4.005333 -2.247336

76 1 0 6.391987 -4.150958 2.401007

77 1 0 6.470465 4.150814 -2.350017

78 7 0 4.061287 0.016721 0.014231

79 16 0 7.233064 -0.012354 0.017929

---------------------------------------------------------------------

**Table S13.** Cartesian coordinates of optimized structures in S_1_ for **2** calculated using Gaussian 09 at the B3LYP/6-31G (d, p) level of theory

Center Number Atomic Number Atomic Type Coordinates (Angstroms)

X Y Z

---------------------------------------------------------------------

1 5 0 -1.831040 -0.025014 -0.005387

2 6 0 -2.583639 -1.243606 -0.685111

3 6 0 -3.582628 -1.033914 -1.689216

4 6 0 -2.261710 -2.598177 -0.341915

5 6 0 -3.974538 0.272821 -2.122546

6 6 0 -4.221513 -2.154583 -2.348034

7 6 0 -2.905339 -3.710641 -1.006664

8 6 0 -1.340994 -2.926029 0.704750

9 6 0 -4.925801 0.465818 -3.094158

10 1 0 -3.510249 1.135453 -1.663923

11 6 0 -5.204098 -1.915068 -3.359190

12 6 0 -3.862657 -3.459492 -1.994804

13 6 0 -2.565699 -5.049886 -0.636470

14 1 0 -0.871748 -2.115766 1.250835

15 6 0 -1.041502 -4.224742 1.039492

16 6 0 -5.554217 -0.641817 -3.723278

17 1 0 -5.198679 1.475601 -3.388293

18 1 0 -5.668907 -2.775704 -3.834633

19 1 0 -4.343931 -4.297856 -2.494455

20 6 0 -1.655084 -5.307151 0.354540

21 1 0 -3.059361 -5.865675 -1.159471

22 1 0 -0.338695 -4.428782 1.843204

23 1 0 -6.302642 -0.473968 -4.493092

24 1 0 -1.413606 -6.330042 0.630958

25 6 0 -2.579906 1.207867 0.653713

26 6 0 -3.606330 1.018517 1.633692

27 6 0 -2.221607 2.555420 0.319497

28 6 0 -4.034980 -0.280221 2.056197

29 6 0 -4.237381 2.152077 2.277911

30 6 0 -2.857082 3.680968 0.969804

31 6 0 -1.268653 2.863532 -0.704369

32 6 0 -5.012967 -0.453927 3.004721

33 1 0 -3.577659 -1.151815 1.607296

34 6 0 -5.248525 1.932327 3.265076

35 6 0 -3.842932 3.449542 1.934542

36 6 0 -2.479695 5.012801 0.609118

37 1 0 -0.804648 2.043815 -1.240886

38 6 0 -0.933557 4.155888 -1.030937

39 6 0 -5.633263 0.666263 3.619518

40 1 0 -5.313895 -1.457921 3.291187

41 1 0 -5.707170 2.802045 3.729878

42 1 0 -4.318464 4.297576 2.423264

43 6 0 -1.540224 5.250892 -0.359704

44 1 0 -2.968512 5.838887 1.120411

45 1 0 -0.210100 4.344872 -1.820019

46 1 0 -6.403362 0.513558 4.370895

47 1 0 -1.271630 6.268652 -0.630225

48 6 0 -0.255083 -0.029822 0.014285

49 6 0 0.512000 -0.808708 -0.894016

50 6 0 0.485897 0.755836 0.938656

51 6 0 1.901049 -0.799396 -0.896800

52 1 0 -0.005579 -1.430399 -1.616820

53 6 0 1.874069 0.769064 0.960846

54 1 0 -0.052204 1.370150 1.652932

55 6 0 2.587647 -0.007679 0.033359

56 1 0 2.460472 -1.394938 -1.613971

57 1 0 2.410140 1.376501 1.687321

58 6 0 4.699527 1.014245 -0.681703

59 6 0 4.724738 -0.884012 0.861822

60 6 0 3.981991 2.152490 -1.097668

61 6 0 6.081241 0.926574 -1.016884

62 6 0 6.109001 -1.172889 0.689912

63 6 0 4.030513 -1.534106 1.900276

64 6 0 4.623733 3.163917 -1.792227

65 1 0 2.929248 2.233631 -0.865107

66 6 0 6.716455 1.959514 -1.711237

67 6 0 6.768702 -2.053784 1.550978

68 1 0 2.976401 -1.338580 2.038719

69 6 0 4.696099 -2.411027 2.739990

70 6 0 5.991678 3.078920 -2.097965

71 1 0 4.049550 4.033344 -2.091894

72 1 0 7.773744 1.877360 -1.941381

73 6 0 6.066156 -2.672129 2.576636

74 1 0 7.827246 -2.246549 1.410067

75 1 0 4.139577 -2.891541 3.536769

76 1 0 6.488874 3.880315 -2.632686

77 1 0 6.582148 -3.351937 3.245196

78 7 0 4.042054 -0.007173 0.012288

79 16 0 7.003194 -0.534996 -0.684859

---------------------------------------------------------------------

**Table S14.** Cartesian coordinates of optimized structures in T_1_ for **2** calculated using Gaussian 09 at the B3LYP/6-31G (d, p) level of theory

---------------------------------------------------------------------

Center Number Atomic Number Atomic Type Coordinates (Angstroms)

X Y Z

---------------------------------------------------------------------

1 5 0 -1.818374 -0.035628 -0.030180

2 6 0 -2.560087 -1.233005 -0.730610

3 6 0 -3.624501 -0.990244 -1.734105

4 6 0 -2.187766 -2.638933 -0.415132

5 6 0 -4.030475 0.296262 -2.073570

6 6 0 -4.241659 -2.093841 -2.413330

7 6 0 -2.814965 -3.726747 -1.107797

8 6 0 -1.254548 -2.944433 0.572724

9 6 0 -5.043965 0.537658 -3.054180

10 1 0 -3.585141 1.153787 -1.584125

11 6 0 -5.228906 -1.851206 -3.362642

12 6 0 -3.823904 -3.443662 -2.104331

13 6 0 -2.448096 -5.035454 -0.807619

14 1 0 -0.768172 -2.146603 1.123356

15 6 0 -0.889259 -4.291608 0.881291

16 6 0 -5.640386 -0.523368 -3.688763

17 1 0 -5.326520 1.561284 -3.279467

18 1 0 -5.696427 -2.694579 -3.864269

19 1 0 -4.293619 -4.272021 -2.626747

20 6 0 -1.473517 -5.325468 0.191850

21 1 0 -2.917464 -5.851844 -1.350293

22 1 0 -0.151729 -4.477105 1.655859

23 1 0 -6.415177 -0.369966 -4.434034

24 1 0 -1.209729 -6.358096 0.400283

25 6 0 -2.584214 1.140607 0.705979

26 6 0 -3.416363 0.871370 1.829508

27 6 0 -2.436880 2.490950 0.272919

28 6 0 -3.629058 -0.451168 2.339006

29 6 0 -4.067168 1.954078 2.536336

30 6 0 -3.103670 3.564471 0.976053

31 6 0 -1.682588 2.854757 -0.892639

32 6 0 -4.420487 -0.687998 3.433171

33 1 0 -3.156407 -1.293076 1.849196

34 6 0 -4.880962 1.667064 3.677096

35 6 0 -3.889978 3.265547 2.091118

36 6 0 -2.955922 4.911029 0.516643

37 1 0 -1.184530 2.079786 -1.463283

38 6 0 -1.568710 4.157361 -1.307127

39 6 0 -5.057822 0.383460 4.116690

40 1 0 -4.560637 -1.706846 3.782445

41 1 0 -5.358927 2.497691 4.189496

42 1 0 -4.384737 4.075861 2.621760

43 6 0 -2.208666 5.205693 -0.591582

44 1 0 -3.461058 5.700045 1.067336

45 1 0 -0.986603 4.390287 -2.194138

46 1 0 -5.679916 0.179574 4.983190

47 1 0 -2.109596 6.232381 -0.931333

48 6 0 -0.244495 -0.023916 -0.023740

49 6 0 0.500609 -0.700681 -1.012138

50 6 0 0.481047 0.672118 0.966351

51 6 0 1.892867 -0.665668 -1.029711

52 1 0 -0.021956 -1.257085 -1.785016

53 6 0 1.873587 0.685797 0.977062

54 1 0 -0.058892 1.209670 1.740315

55 6 0 2.586733 0.023566 -0.030813

56 1 0 2.456647 -1.175160 -1.804611

57 1 0 2.416216 1.217039 1.753172

58 6 0 4.676588 1.170269 -0.616907

59 6 0 4.725970 -0.895889 0.746038

60 6 0 4.000305 2.383126 -0.822407

61 6 0 6.034363 1.098603 -0.985902

62 6 0 6.088399 -1.162122 0.505739

63 6 0 4.094694 -1.582109 1.795398

64 6 0 4.656309 3.484071 -1.373327

65 1 0 2.952994 2.465392 -0.560942

66 6 0 6.693706 2.217206 -1.496475

67 6 0 6.795115 -2.048450 1.319145

68 1 0 3.045211 -1.407759 1.995713

69 6 0 4.798234 -2.498628 2.576922

70 6 0 6.007052 3.413200 -1.704723

71 1 0 4.101149 4.404147 -1.529475

72 1 0 7.746266 2.134968 -1.750404

73 6 0 6.152737 -2.731161 2.351883

74 1 0 7.849646 -2.215205 1.121033

75 1 0 4.277570 -3.021614 3.373507

76 1 0 6.523039 4.272064 -2.121666

77 1 0 6.705506 -3.434290 2.966521

78 7 0 4.024433 0.034149 -0.067021

79 16 0 6.892378 -0.460540 -0.919966

---------------------------------------------------------------------

# References

[S1] Becke, A. D., A new mixing of Hartree–Fock and local density‐functional theories. *The Journal of Chemical Physics* **1993,** *98* (2), 1372-1377.

[S2] Frisch, M. J.; Trucks, G. W.; Schlegel, H. B.; Scuseria, G. E.; Robb, M. A.; Cheeseman, J. R.; Scalmani, G.; Barone, V.; Mennucci, B.; Petersson, G. A.; Nakatsuji, H.; Caricato, M.; Li, X.; Hratchian, H. P.; Izmaylov, A. F.; Bloino, J.; Zheng, G.; Sonnenberg, J. L.; Hada, M.; Ehara, M.; Toyota, K.; Fukuda, R.; Hasegawa, J.; Ishida, M.; Nakajima, T.; Honda, Y.; Kitao, O.; Nakai, H.; Vreven, T.; Montgomery, J. A., Jr.; Peralta, J. E.; Ogliaro, F.; Bearpark, M.; Heyd, J. J.; Brothers, E.; Kudin, K. N.; Staroverov, V. N.; Kobayashi, R.; Normand, J.; Raghavachari, K.; Rendell, A.; Burant, J. C.; Iyengar, S. S.; Tomasi, J.; Cossi, M.; Rega, N.; Millam, J. M.; Klene, M.; Knox, J. E.; Cross, J. B.; Bakken, V.; Adamo, C.; Jaramillo, J.; Gomperts, R.; Stratmann, R. E.; Yazyev, O.; Austin, A. J.; Cammi, R.; Pomelli, C.; Ochterski, J. W.; Martin, R. L.; Morokuma, K.; Zakrzewski, V. G.; Voth, G. A.; Salvador, P.; Dannenberg, J. J.; Dapprich, S.; Daniels, A. D.; Farkas, O.; Foresman, J. B.; Ortiz, J. V.; Cioslowski, J.; Fox, D. J. Gaussian 09, Revision A.02; Gaussian, Inc., Wallingford, CT, 2009.

[S3] Becke, A. D., Density-functional exchange-energy approximation with correct asymptotic behavior. *Physical Review A* **1988,** *38* (6), 3098-3100. d) Lee, C.; Yang, W.; Parr, R. G., Development of the Colle-Salvetti correlation-energy formula into a functional of the electron density. *Physical Review B* **1988,** *37* (2), 785-789.
