# Supplementary material for: Design, Synthesis, and Temperature-Driven Molecular Conformation-Dependent Delayed Fluorescence Characteristics of Dianthrylboron-Based Donor–Acceptor Systems
Source: Front Chem. 2020 Oct 9;8:541331. doi: 10.3389/fchem.2020.541331 (PMC7581868; doi:10.3389/fchem.2020.541331)
Supplement: Supplementary file 3 [file Data_Sheet_2.PDF]

checkCIF (basic structural check) running

Checking for embedded fcf data in CIF ...
Found embedded fcf data in CIF. Extracting fcf data from uploaded CIF, please wait . . .

checkCIF/PLATON (basic structural check)

You have not supplied any structure factors. As a result the full set of tests cannot be run.

THIS REPORT IS FOR GUIDANCE ONLY. IF USED AS PART OF A REVIEW PROCEDURE FOR PUBLICATION, IT SHOULD NOT REPLACE THE EXPERTISE OF AN EXPERIENCED CRYSTALLOGRAPHIC REFEREE.

You have not supplied any structure factors. As a result the full set of tests cannot be run.

No syntax errors found.
Please wait while processing ....

CIF dictionary
Interpreting this report

Datablock: An2BNPh2

```
Bond precision:       C-C = 0.0020 A              Wavelength=0.71073
Cell:       a=11.3417(7)      b=11.6036(8)      c=13.8519(9)
            alpha=99.345(3)   beta=108.237(4)   gamma=114.742(3)
Temperature: 100 K
```

	Calculated	Reported
Volume	1478.73(19)	1478.73(17)
Space group	P -1	P -1
Hall group	-P 1	-P 1
Moiety formula	C40 H28 B N [+ solvent]	?
Sum formula	C40 H28 B N [+ solvent]	C40 H28 B N
Mr	533.44	533.44
Dx,g cm-3	1.198	1.198
Z	2	2
Mu (mm-1)	0.068	0.068
F000	560.0	560.0
F000'	560.20	
h,k,lmax	16,16,19	16,16,19
Nref	9131	9033
Tmin,Tmax	0.992,0.996	
Tmin'	0.989	

```
Correction method= Not given
Data completeness= 0.989          Theta(max)= 30.613
R(reflections)= 0.0506( 6794)          wR2(reflections)= 0.1470( 9033)
S = 1.050               Npar= 379
```

The following ALERTS were generated. Each ALERT has the format
test-name_ALERT_alert-type_alert-level.
Click on the hyperlinks for more details of the test.

Alert level G

```
PLAT152_ALERT_1_G The Supplied and Calc. Volume s.u. Differ by ...          2 Units
PLAT605_ALERT_4_G Largest Solvent Accessible VOID in the Structure        154 A**3
PLAT869_ALERT_4_G ALERTS Related to the Use of SQUEEZE Suppressed           ! Info
```

```
0 ALERT level A = Most likely a serious problem - resolve or explain
0 ALERT level B = A potentially serious problem, consider carefully
0 ALERT level C = Check. Ensure it is not caused by an omission or oversight
3 ALERT level G = General information/check it is not something unexpected

1 ALERT type 1 CIF construction/syntax error, inconsistent or missing data
0 ALERT type 2 Indicator that the structure model may be wrong or deficient
0 ALERT type 3 Indicator that the structure quality may be low
2 ALERT type 4 Improvement, methodology, query or suggestion
0 ALERT type 5 Informative message, check
```

It is advisable to attempt to resolve as many as possible of the alerts in all categories. Often the minor alerts point to easily fixed oversights, errors and omissions in your CIF or refinement strategy, so attention to these fine details can be worthwhile. In order to resolve some of the more serious problems it may be necessary to carry out additional measurements or structure refinements. However, the purpose of your study may justify the reported deviations and the more serious of these should normally be commented upon in the discussion or experimental section of a paper or in the "special_details" fields of the CIF. checkCIF was carefully designed to identify outliers and unusual parameters, but every test has its limitations and alerts that are not important in a particular case may appear. Conversely, the absence of alerts does not guarantee there are no aspects of the results needing attention. It is up to the individual to critically assess their own results and, if necessary, seek expert advice.

Publication of your CIF in IUCr journals

A basic structural check has been run on your CIF. These basic checks will be run on all CIFs submitted for publication in IUCr journals (*Acta Crystallographica*, *Journal of Applied Crystallography*, *Journal of Synchrotron Radiation*); however, if you intend to submit to *Acta Crystallographica Section C* or *E* or *IUCrData*, you should make sure that full publication checks are run on the final version of your CIF prior to submission.

Publication of your CIF in other journals

Please refer to the *Notes for Authors* of the relevant journal for any special instructions relating to CIF submission.

PLATON version of 22/12/2019; check.def file version of 13/12/2019

Datablock An2BNPh2 - ellipsoid plot

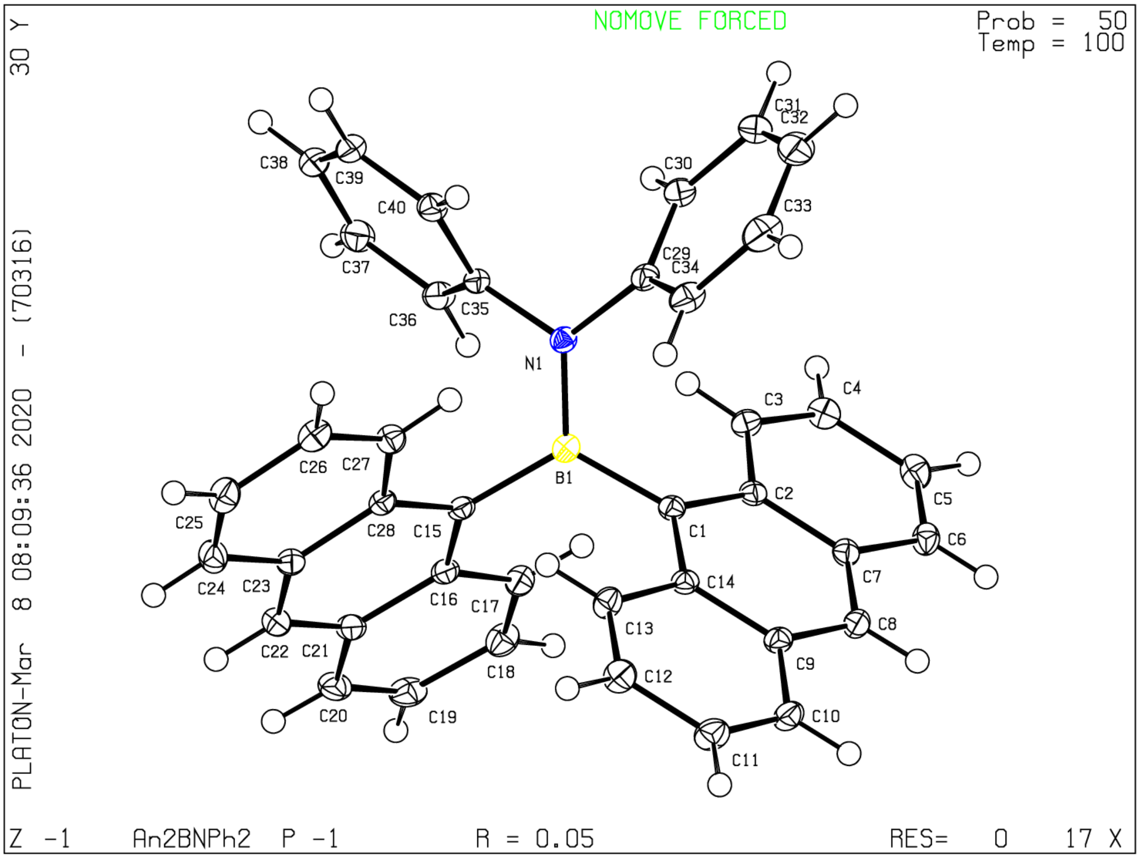

Download CIF editor (publCIF) from the IUCr
Download CIF editor (enCIFer) from the CCDC
Test a new CIF entry
